# A Combination of Transcriptional and MicroRNA Regulation Improves the Stability of the Relative Concentrations of Target Genes

**DOI:** 10.1371/journal.pcbi.1003490

**Published:** 2014-02-27

**Authors:** Andrea Riba, Carla Bosia, Mariama El Baroudi, Laura Ollino, Michele Caselle

**Affiliations:** 1 Department of Physics and INFN, University of Torino, Torino, Italy; 2 Human Genetics Foundation (HuGeF), Torino, Italy; 3 National Research Council (CNR), Institute of Informatics and Telematics (IIT) and Institute of Clinical Physiology (IFC), Laboratory for Integrative System Medicine (LISM), Pisa, Italy; University of Southern California, United States of America

## Abstract

It is well known that, under suitable conditions, microRNAs are able to fine tune the relative concentration of their targets to any desired value. We show that this function is particularly effective when one of the targets is a Transcription Factor (TF) which regulates the other targets. This combination defines a new class of feed-forward loops (FFLs) in which the microRNA plays the role of master regulator. Using both deterministic and stochastic equations, we show that these FFLs are indeed able not only to fine-tune the TF/target ratio to any desired value as a function of the miRNA concentration but also, thanks to the peculiar topology of the circuit, to ensure the stability of this ratio against stochastic fluctuations. These two effects are due to the interplay between the direct transcriptional regulation and the indirect TF/Target interaction due to competition of TF and target for miRNA binding (the so called “sponge effect”). We then perform a genome wide search of these FFLs in the human regulatory network and show that they are characterized by a very peculiar enrichment pattern. In particular, they are strongly enriched in all the situations in which the TF and its target have to be precisely kept at the same concentration notwithstanding the environmental noise. As an example we discuss the FFL involving E2F1 as Transcription Factor, RB1 as target and miR-17 family as master regulator. These FFLs ensure a tight control of the E2F/RB ratio which in turns ensures the stability of the transition from the G0/G1 to the S phase in quiescent cells.

## Author Summary

Gene expression is controlled by a complex network of regulatory interactions which may be organized in two complementary subnetworks: the transcriptional one, mediated by Transcription Factors (TF), and the post-transcriptional one, in which a central role is played by microRNAs. In this paper we add a further step in the study of synergistic role of these layers of regulation: a stable fine tuning of the relative expression of target genes is obtained by a combination of transcriptional and post-transcriptional interactions, and such a combination ensures robustness against stochastic fluctuations. We show that optimal fine tuning is reached when the microRNA plays the role of master regulator and one of its targets is a TF which regulates the other microRNA targets. This combination defines a new class of feed-forward loops. We show that such circuitries are strongly enriched when the TF and its targets have to be precisely kept at the same concentration notwithstanding the environmental noise. We complete our analysis with a detailed description, using both deterministic and stochastic equations, of the steady state concentrations of the genes involved in the motifs as a function of the miRNA concentration and of the miRNA-target interaction strength.

## Introduction

The interplay between transcriptional and post-transcriptional regulation attracted much interest in the past few years [1]. As in the purely transcriptional regulatory network [2], motifs belonging to such mixed layer of interaction have been identified [3]–[6] and mathematically characterized [4], [5], [7]–[9]. MicroRNAs (miRNAs), small non-coding RNAs which post-transcriptionally regulate gene expression, play a pivotal role in these circuitries. So far the attention was mainly devoted to circuits in which miRNAs have only an auxiliary role. This is the case for instance of the miRNA-mediated Feed Forward Loop (FFL) [4], [5], [7], [8] or the miRNA mediated self-loop [9]. However, several important biological processes are actually controlled by miRNAs which play themselves the role of master regulators. The corresponding network motifs show a remarkable degree of topological enrichment in the mixed regulatory network [10], [11]. A major reason of interest in this type of circuits is the so called “sponge effect” [12], [13], i.e. the appearance of indirect interactions among targets due to competition for miRNA binding.

**Figure 1:**
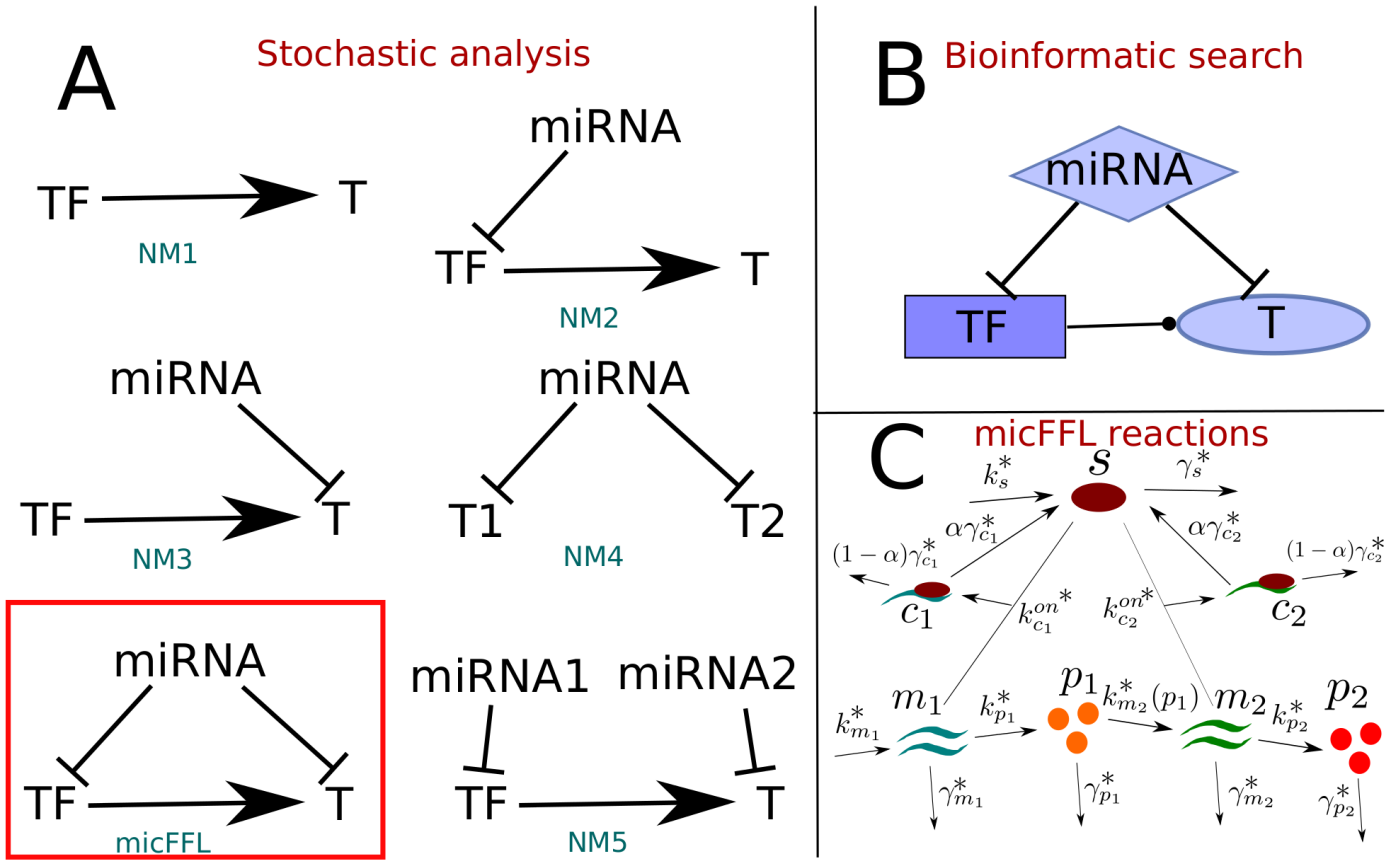
A. Schematic description of the circuits discussed in the paper. NM1: direct regulation; NM2: open motif in which the microRNA regulates only the transcription factor; NM3: open motif in which the microRNA regulates only the target; NM4: Open motif in which the microRNA regulates both the TF and the target but the TF-target link is missing; NM5, open motif in which two different microRNAs regulate separately the TF and the target. In the box we show the activactory micFFL whose deterministic and stochastic behavior we studied in the paper. **B**. Schematic view of the general miRNA controlled Feed Forward Loops (combining both activactory and repressive TF-target interactions) mined in the bioinformatic analysis discussed in the paper. **C**. Schematic description of the chemical reactions which must be taken into account to describe the miRNA-mediated feedforward loop with a miRNA-target titrative interaction.

In [10] analysis of data from the *Encyclopedia of DNA Elements (ENCODE)* project revealed that two distinct classes of miRNA-controlled circuits were particularly enriched in the network. In the first class miRNAs target two interacting genes (which for example can dimerize). MiRNAs belonging to the second class target two transcription factors (TFs) which both regulate the same gene, one as proximal and one as distal regulator. This same topology was found to be over-represented in human glioblastoma combining bioinformatical analysis and expression data [11]. Both these examples suggest a role of miRNAs in ensuring the stability and fine-tuning of the relative concentration of their targets. The topological enrichment is further magnified if one selects those motifs in which the two targets are linked by a transcriptional regulation (see Figure 1B). The resulting network motif is a FFL in which a miRNA regulates a TF and together with it one or more target (T) genes. In the following we shall denote these circuitries as “miRNA-controlled FeedForward loops” (micFFL).

An interesting feature of the micFFL is that it is the simplest motif in which a TF regulates its target simultaneously with direct (transcriptional) and indirect (mediated by the sponge effect) regulatory interactions. Depending on the sign of the transcriptional regulation this combination can be coherent or incoherent and may have very interesting functional roles. In this paper we address the case of an activatory transcriptional regulation (see the left bottom motif of Figure 1A). The transcriptional version of this circuit has been analyzed by several authors [14], [15]. The circuit is able to perform a few important functions able to enhance the coordination of the targets. At the same time, targets' coordination may represent a too strong linkage, thus decreasing the overall flexibility of the network. This non-trivial behavior could be the reason of the quite peculiar pattern of topological enrichment we observe. Our main goal will be to quantitatively study these functions, to fix the range of parameters in which they occur and, possibly, to understand their role within the regulatory network as a whole.

We address the model both at the deterministic and at the stochastic level. In order to quantify the behavior of the various molecular species involved, we then compare the micFFL with four different miRNA-mediated regulatory topologies involving the same players (one miRNA, one TF and one T). In all cases the miRNA-target interaction will be modelled via a titration-like mechanism, i.e. we assume that miRNA and target may only interact by forming a complex which eventually degrades [16]. After degradation of the complex the miRNA may be recycled. This choice, at the basis of the sponge effect, will play a major role in our analysis. In fact it has been shown that titration-like mechanisms entail, among other properties, cross-talk and statistical correlation between different targets in competition for the same group of molecules [17]–[22]. We show that the sponge interaction between TF and T induces a statistical correlation between them much stronger than in case of simple transcriptional regulation. Moreover, this linkage holds for a range of miRNA concentrations larger than in the other circuits and reaches its maximum exactly when TF and T show the highest degree of stochastic fluctuations. Altogether these observations support the general picture of miRNAs as homeostasis controllers [8], [13], with different roles depending on the particular topologies they are embedded in. In particular, coherent micFFL could be useful in situations in which TF and T concentrations have to be precisely kept at the same ratio notwithstanding the environmental noise. In the last section we discuss a prototypical example of this situation, i.e. the micFFL involving E2F1 as TF, RB1 as T and a set of miRNAs (miR-106a, miR-106b, miR-17, miR-20a and miR-23b) as master regulators. This circuit is involved in the fine-tuned control of the transition from the G0/G1 to S phase in the cell cycle. This transition is triggered by the difference in concentration of the two targets. We shall argue below that the micFFLs controlling the two genes were selected by evolution exactly to avoid accidental triggering of the transition due to uncorrelated stochastic fluctations of the two proteins. The comparison with the other topologies shows that the simple loss or addition of one of the interactions in the loop could destroy this linkage and lead to pathological behaviors.

## Results

### Bioinformatic search of micFFL in the human regulatory network

A detailed description of our procedure is reported in the Material and Methods section, we only report here the main steps. Briefly, we constructed a list of putative micFFLs combining miRNA-T and TF-T regulatory interactions obtained as follows. For the miRNA-T side we integrated information obtained from four freely available databases of miRNA-T interactions, chosen so as to have the widest possible spectrum of different prediction strategies: doRiNA [23], microRNA.org [24], TargetScan [25] and PITA [26]. We selected as potential targets only transcripts corresponding to protein-coding genes completely annotated in Ensembl 68 [27]. For the TF-T side we used two different strategies. In the first one we selected the TFs contained in the JASPAR database [28], [29] and used the corresponding Position Frequency Matrix (PFM) to construct a search algorithm for transcription factor binding sites (TFBS) within the target promoter regions. We found in this way a total of 948125 interactions. In the second approach we simply used as signatures of TF-T interactions the ChIP-seq results of the ENCODE project [10]. Combining together the results of the five cell lines of the ENCODE project we obtained a total of 45328 TF-T interactions. We obtained in this way a total of 75933600 micFFLs with miRNA-T interaction confirmed by at least one database in the JASPAR case and a total of 2426300 micFFLs in the ENCODE case. We chose this twofold strategy to construct the TF-T side of our network so as to have an independent check for the enrichment analysis. In fact with the ENCODE list, based on ChIP-seq experiments, we expect to have a smaller rate of false positives results with respect to a purely bioinformatic approach. At the same time, using only the ENCODE list could induce a statistical bias in the results due to the fact that ChIP-seq experiments were performed only for a small subset of TFs, selected for their particular biological relevance. This could in principle create problems when performing a topological enrichment analysis. For this reason we chose to supplement this analysis with an alternative procedure which has exactly the opposite features: it is an unbiased genome-wide bioinformatic search from sequence information only, with no reference to experimental results. The obvious drawback of this second approach is the possible presence of several false positives. As we shall see below our enrichment analysis gives similar enrichment scores for both strategies thus strongly supporting the reliability of results.

### Enrichment test

**Figure 2:**
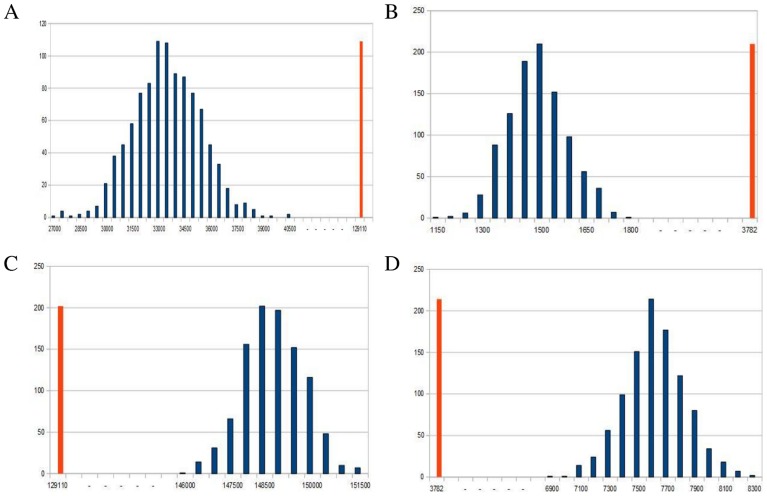
A. Randomization of miRNA-target links. Distribution of the number of FFLs for 1000 simulations obtained with JASPAR TFs list and confirmed by at least 4 miRNA databases (Z = 49,4). **B**. Randomization of miRNA-target links. Distribution of the number of FFLs for 1000 simulations obtained with ENCODE TFs list and confirmed by at least 4 miRNA databases (Z = 23,3). **C**. Randomization of TF-target links. Distribution of the number of FFLs for 1000 simulations obtained with JASPAR TFs list and confirmed by at least 4 miRNA databases (Z = −20,8). **D**. Randomization of TF-target links. Distribution of the number of FFLs for 1000 simulations obtained with ENCODE TFs list and confirmed by at least 4 miRNA databases (Z = −18,1).

In order to minimize the number of false positives we selected only micFFLs in which both the miRNA-TF and the miRNA-T links were confirmed by all the four databases. This choice reduced the number of micFFLs to 129110 in the JASPAR case and 3782 in the ENCODE case. Since the links of the loop are not on the same ground we performed a topological enrichment analysis by random reshuffling *separately* the post-transcriptional and transcriptional links of the micFFL. First we randomized miRNA-T links keeping TF-T links fixed. We made 1000 simulations. For each miRNA we extracted random targets within the Ensembl 68 list of known protein-coding transcripts keeping fixed the number of targets (i.e. keeping unchanged the outdegree of the miRNA nodes). We performed the simulation both for the JASPAR and the ENCODE lists of TF-T interactions and evaluated a z-score. The z-score was defined, as usual, as 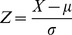, where 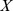 is the number of micFFL in the real network, while 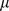 and 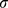 are the mean and the standard deviation of the same quantity in the sample of 1000 simulated networks. In both cases we found very high values of the z-score (see Figures 2A–B): 49.4 for Jaspar and 23.3 for Encode. We then randomized TF-T links, keeping the miRNA-T links unchanged. Also in this case we kept fixed the outdegree of the TF nodes of the network and perfomed the reshuffling both for the JASPAR and ENCODE lists. Remarkably enough we found this time in both cases a very strong *negative* enrichment (see Figures 2C–D), with z-score values of the same magnitude of previous case: −20.8 for JASPAR and −18.1 for ENCODE. The simplest explanation of this very peculiar behavior is that miRNAs seem to target preferentially TFs (this largely explains the large positive enrichment in the first reshuffling test) but at the same time the particular topology of the micFFL seems to be strongly selected against by evolution and is preferentially *avoided* within the network. These observations make micFFLs a very interesting subject of study. It seems that its particular topology induces very strong constraints on the behavior of its targets and might be in general dangerous for the performances of the network. Consequently, when one of this circuits is actually realized in the network it is certainly not by chance and it is likely to play a well precise functional role. The remaining part of this paper will be mainly devoted to understand this issue. It is very interesting to observe that the enrichment pattern is essentially the same both in the JASPAR and in the ENCODE cases. Since the two TF datasets have a rather small overlap (only 38 TFs are in common) and the approaches to detect regulatory interactions are completely independent, the similarity of the two enrichment patterns is a strong evidence of their reliability and robustness. Finally it is worthwhile to stress that this very peculiar enrichment pattern almost disappears and would escape detection if one simultaneously permutes both transcriptional and post-transcriptional interactions due to the compensation between positive and negative enrichments. According to the standard classification of FFLs (see for instance [30], [31]), the micFFL is a coherent C2 FFL if the TF protein positively regulates its target gene, or an incoherent I2 type if the TF protein negatively regulates its target. It would be very interesting to perform separate enrichment tests for the coherent and the incoherent cases but unfortunately neither the JASPAR nor the ENCODE databases contain information on the sign of the TF regulation.

### Putative functions of micFFLs

It has been recently shown that microRNAs can generate thresholds in target gene expression [16] which in turn may induce non-linear relations between protein and transcript concentrations. In the same paper it was also pointed out that gene expression shows large cell-to-cell fluctuations in a population of identically prepared cells. We find that similar threshold effects are also present in the TF and T of micFFLs whose relative concentrations can be fine-tuned to any desired value as function of miRNA concentration. In particular, the peculiar topology ensures a tight control of stochastic fluctuations of this ratio and the noise reduction is maximal exactly in proximity to the threshold region. We perform the analysis of the circuit in two main steps (deterministic and stochastic) concentrating on the behavior of the ratio 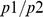 for the concentration of two targets. The robustness of this ratio against stochastic fluctuations is one of the main reasons of interest on this circuit and will be the main issue of the stochastic analysis. A more intuitive enquiry (a “logical approximation”) is present in the Supplementary Information (S1). In order to discuss the functional properties of the micFFL we compare it with five “null models” obtained eliminating miRNA-TF and/or miRNA-T interactions. We can thus identify which properties are direct consequences of the miRNA interaction (as the threshold effect) or are a peculiar consequence of the micFFL topology (as the noise reduction).

The simplest null model is represented by the direct regulation TF→T without miRNAs (NM1). Comparison with NM1 shows the effect of switching-on the miRNA in our circuit. Two other important null models are those circuits in which we only keep the miRNA-TF interaction (NM2) or the miRNA-T interaction (NM3) (see Figure 1A). Finally, we analyze the circuit with one miRNA regulating separately the two targets T1 and T2 (NM4) and the open circuit in which two independent miRNAs regulate TF and T respectively (NM5) (see Figure 1A). These circuits are themselves very interesting. In particular NM4 was widely studied in the past few years to model bacterial small RNA (sRNA)/target interaction [18], [19]. More recently it was also discussed in the framework of a miRNA/target interaction network [20]–[22] as an example of the sponge effect. A byproduct of our analysis will be the discussion of few interesting features of these null models.

### Deterministic analysis

The micFFL is described by the following set of equations:
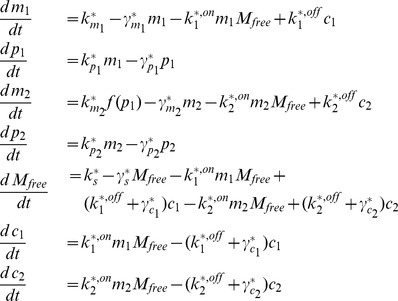
(1)where 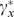 denotes the degradation constant of the molecular species 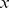 and 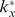 the corresponding production rate, 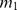 and 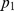 the concentration of mRNA and protein for the TF and 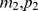 those for the target. We then redefine the parameters dividing them by the target protein degradation rate 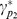 in order to have dimensionless values. The system thus becomes:
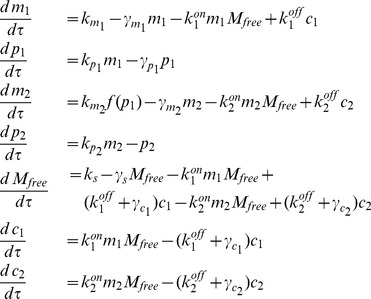
(2)where 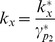 are the rescaled transcription or translation rates, 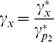 the rescaled degradation rates and 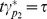 the rescaled time. Following [16] we assumed that miRNA can interact with target mRNA 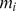 by forming a complex 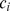 with it. The 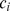 stability is determined by the costants 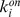, 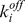 and by the concentration of unbound miRNA 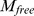. 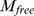 is related to the total concentration of miRNA 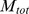 by the relation:
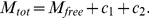
(3)In the following 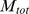 is an external input of the circuit. The transcriptional regulation of 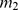 is described by the activatory Hill function
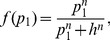
(4)with Hill coefficient 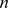 and activation coefficient 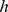. A section of the Supporting Information S1 is devoted to discuss the explicit introduction of the promoter state dynamics for the target gene. The equations describing the null models introduced above (discussed in detail in Supporting Information S1) can be easily obtained from Eq.s 2 eliminating some of the molecular species and/or interactions.

**Figure 3:**
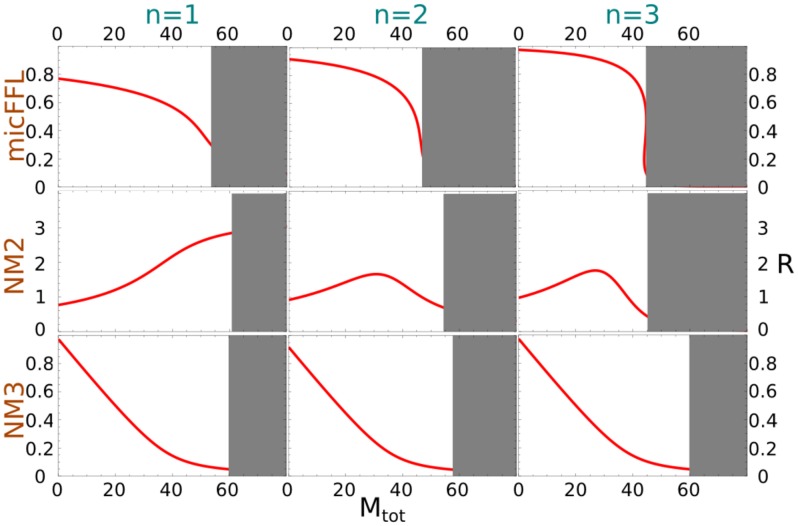
The ratio of the target and TF concentrations as a function of 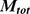 for the micFFL and the NM2 and NM3 null models for three values 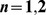 and 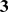 of the Hill exponent.

The steady state solution of Eq.s (2) can be written in a simple way as a function of 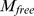. Introducing
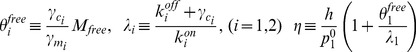
(5)we can write
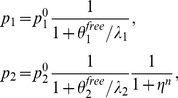
(6)where 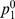 and 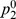 denote the asymptotic values of 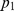 and 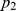 in absence of miRNAs. The Hill function is at saturation, i.e. 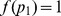 (similarly for 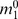 and 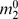), so that 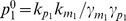 and 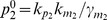. From these equations we obtain the ratio 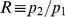 as a function of 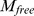:
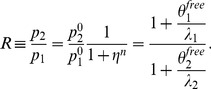
(7)It would be interesting to obtain the same ratio as a function of 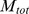 instead of 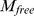. 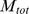 can be obtained from 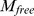, 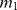 and 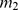


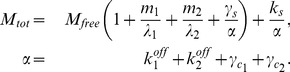
(8)The dependence on 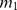 and 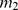 makes it difficult to write the ratio explicitly in terms of 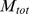, but it can be easily obtained numerically. We plot 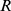 as a function of 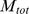 in Figure 3 in the limit 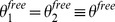 and 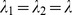 for 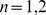 and 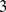. We plot for comparison the same ratio for the null models NM2 and NM3. The shadowed portions of the plots denote the regions in which either 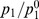 or 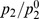 is less than 0.05, i.e where the miRNA concentration is so high that one of the proteins (or both) is almost absent. As miRNA concentration increases, 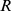 can be tuned from 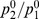 down to less than 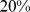 of its orginal value. The shape of the 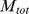 dependence and the minimum value of 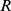 strongly depend on the Hill coefficient. It is interesting to observe that also NM2 and NM3 allow to fine tune 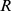 essentially to any desired value. These two models represent the limiting situations which one would obtain when 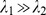 or 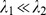.

### Stochastic analysis

As in the previous section, we assume a titrative miRNA-target interaction and an activatory Hill function for the TF-dependent target transcription rate. The molecular species we considered are transcripts for miRNAs (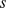), transcription factor (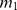) and target (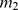), proteins for transcription factor (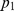) and target (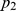), and the complexes the miRNA can form when bound to 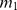 or 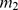 (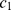 and 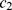 respectively). The parameters are defined as in (1). The chemical reactions involved in the circuit are schematically reported in Figure 1C. The corresponding master equation, setting 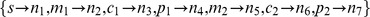, is
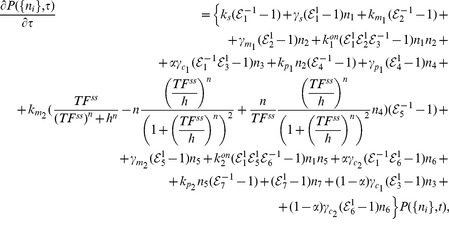
(9)where 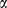 denotes the probability of miRNA recycling and 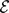 is the step-operator 
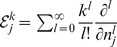
. As in [8], [9] we linearized the Hill function around the steady state value 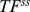 (see Supporting Information S1 for further details). The analogous equations for the null models are discussed in Supporting Information S1.

We are interested in evaluating the linear correlation coefficients 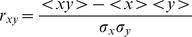, which measures how much two variables are linearly dependent.

This quantity can be evaluated in general for any pair of molecular species, but we are in particular interested in the correlation between 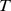 and 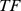. To estimate it we need the first two moments of the probability distribution 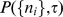. Due to the complexity of the master equation this cannot be done analytically not even by linearizing the target transcription rate, thus we decided to approach the problem in the framework of the linear noise approximation [32]. In this framework it is straightforward to obtain the covariance matrix of the system directly from its macroscopic description [17] and thus have approximate expressions for the first two moments of 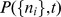. We performed a set of Gillespie simulations on the model in order to quantify the error due to the linear noise approximations. Details on all these calculations can be found in SI.

We made an effort to present all the results in terms of potentially measurable parameters, such as miRNA number of molecules and miRNA-target interaction strenght 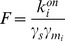 [18] (where 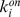, 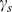, 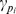 and 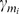 are defined as above). The other parameters take physiological values (and a section of SI is devoted to a brief stability analysis over their fluctuations). We estimate the parameters' order of magnitude via the transcription, translation and degradation rates found in [33] and Bionumbers database [34]. To test our choice, we checked whether the steady state concentrations have realistic values. In order to understand the peculiar properties of micFFL we compared it with the null models NM3,NM4 and NM5. Given the large number of free parameters, such a comparison is not straightforward. Our strategy was to maintain equal all the corresponding parameters in the four models and then compare all of them with the direct regulation (NM1), i.e. with the situation in which the miRNA is switched-off.

### miRNA-controlled feedforward loop increases TF-T statistical correlation

**Figure 4:**
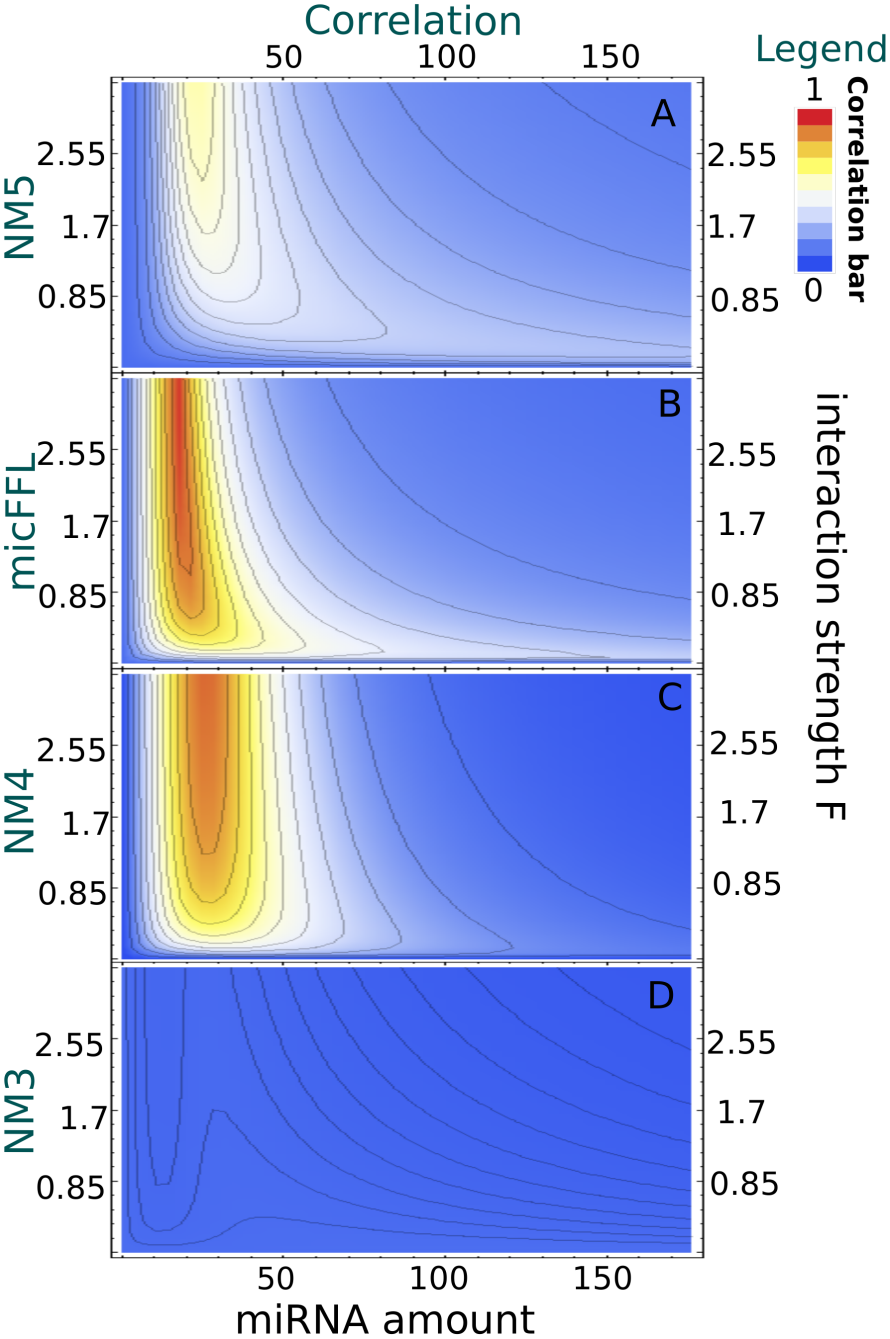
Heat map of the correlation 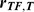 for the micFFL and NM3,NM4 and NM5 Null Models. In each plot the values of 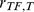 is mapped as a function of the miRNA concentration and of the interaction strength 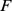. While for NM3 and NM5 the fluctuation of TF and T are almost uncorrelated, both NM4 and the micFFL show a well defined region of large correlation. This correlation occurs for rather low miRNA concentrations and for almost any value of the miRNA-mRNA interaction strength.

We report in Figure 4 our estimates for the correlation coefficient between TF and T (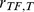) for micFFL, NM3, NM4 and NM5. Both micFFL and NM4 show wide regions of the parameter space in which TF and T are strongly correlated while for NM3 and NM5 the correlation is almost negligible. This trend is an unequivocal consequence of the titration which establishes an indirect interaction between transcripts in competition for binding the same miRNA. We think that the enhanced statistical correlation of targets is the ultimate reason for the generic enrichement observed in [10], [11] for this type of motif: targets in physical interaction are likely to require stable stoichiometric ratios.

Comparing Figure 4B with Figure 4C we see that this correlation is further enhanced in the micFFL by the transcriptional link between TF and T. This enhancement is due to the interplay between the direct link TF-T and the indirect miRNA-mediated TF-T link. Figure 4A reproduces the situation in which two independent miRNA genes (with the same kinetic parameters of miFFLC) target TF and T independently (NM5). The TF-T correlation profile results here from the bare fact that TF is an activator of T (direct link). The NM4 case instead (Figure 4C) could be a proxy for the indirect effect alone. In SI, a more detailed comparison between NM4 and micFFL was done. Both circuits increase greatly the correlation, but micFFL, thanks to the regulatory link, reaches always higher values of correlation. The union of NM5 and NM4 correlation profiles is indeed very similar to the miFFLC one. For completeness we also analyzed the case in which the link miRNA-TF is lacking (NM3). Here again the correlation profile is due only to the direct TF-T connection. The heat-map does not show appreciable differences exploring the parameter space and the TF-T correlation values are almost everywhere comparable with that of a simple direct regulation.

### Threshold effects in micFFL and NM4 motifs

Titrative interactions may induce threshold effects among the interacting molecules and system hypersensitivity in proximity to the threshold [16]. In the particular cases of micFFL and NM4 this effect involves three molecular species simulateneously (miRNAs, TFs and Ts) and gives rise to a very peculiar behavior. In NM4, when the amount of miRNA is similar to the amount of mTF and mT, a small fluctuation in even only one of their concentrations could be enough to move the system in the protein expressed or repressed phase. Right in this condition of near-equimolarity of competing species the system is hypersensitive in changing of control parameters, as miRNA or targets transcription rates [35]. The threshold is indeed determined by the model kinetic parameters and in the limit of strong interaction strength (high value of F) can be located in 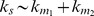 [17], [18], [20]. In miFFLC the situation is similar, but the direct link between TF and T increases the effective target transcription rate thus shifting the threshold toward a miRNA transcription rate higher than in NM4. As a consequence, also the hypersensitivity region shifts its right-boundary.

### Switch-on and switch-off response times

**Figure 5:**
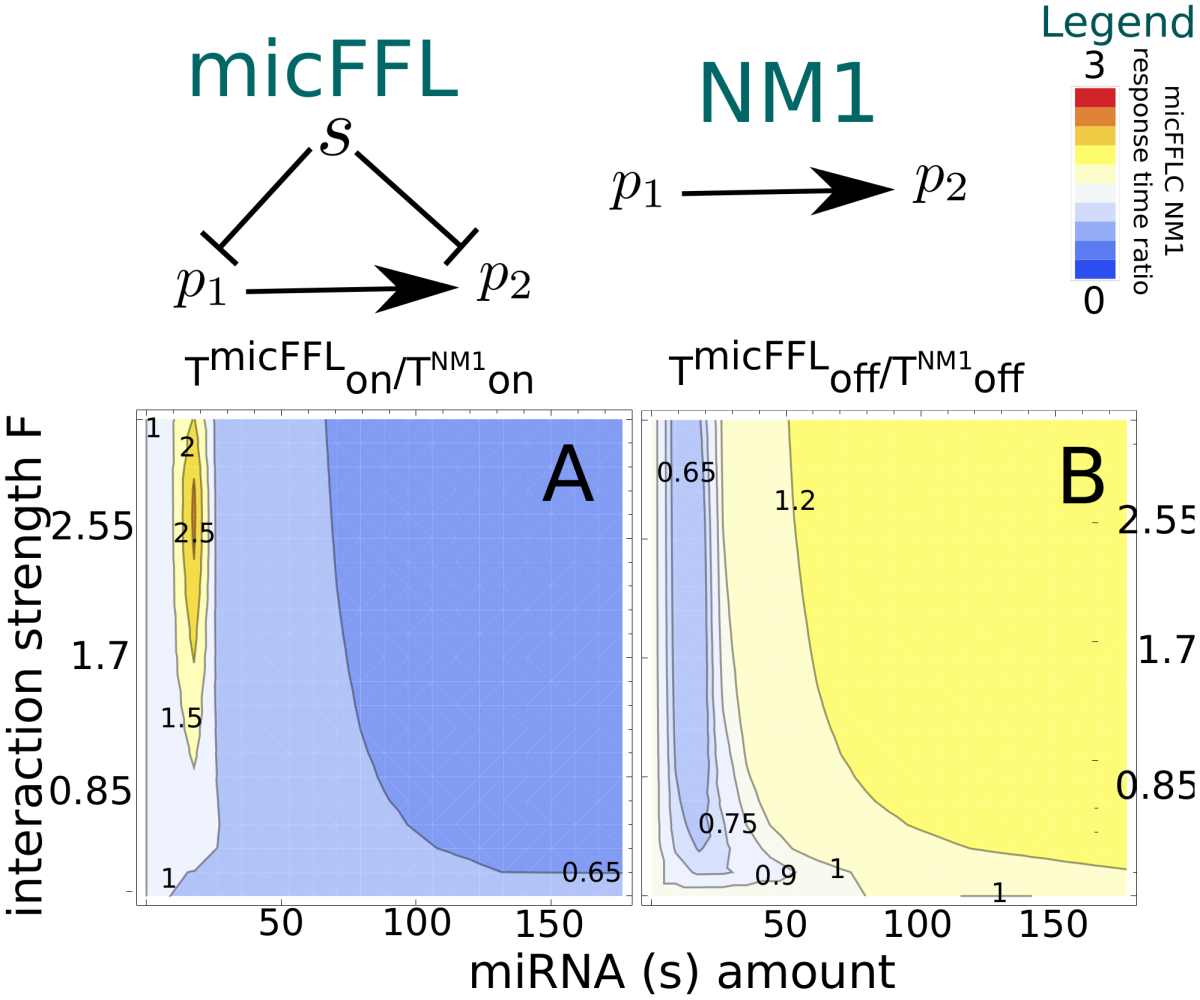
Comparison of switch-on (A) and switch-off (B) response times between micFFL and direct regulation (NM1).

In several cases the price to pay to be able to tightly control protein concentrations is a slowing down of response times. Response time is defined as the time the target protein needs to reach half of the value of its final (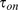) or initial (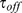) steady state upon sudden activation or deactivation of TF transcription, that is 
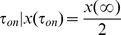
 and 
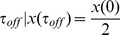
. The behaviour of response times in purely transcriptional FFLs was studied in detail in the past few years [30], [31], the aim of this section is to address the same issue in the micFFL. To this end we evaluated the switch-on and switch-off response times of the target in micFFL and compared them with the analogous quantities in NM1. We fixed the parameters of micFFL so as to have the same steady state concentrations both for TF and T. The remaining free parameters are the miRNA amount and interaction strength. We can thus study the change in the switch-on and switch-off response times as a function of these quantities. The results are reported in Figure 5. As it is easy to see the response times are always of the same order of magnitude of those of NM1. In particular as the miRNA concentration increases the switch-on time decreases and, for physiological concentrations of the target, reaches the steady state *faster* than in absence of miRNA. The efficiency of the miRNA plays only a minor role in this trend. The opposite is true for the switch-off time which shows a moderate increase while increasing miRNA concentration and are instead strongly depressed for low miRNA concentrations. It would be very interesting to extend our analysis to keep into account also a possible self-regulatory interaction of the TF, which is a quite common situation in the human regulatory network. A detailed study of this more complex motif is beyond the scope of the present paper, but we expect that the main effect of the self-regulatory interaction should be to induce a change in the switch-on and switch-off response times. The role of self-regulation in tuning response times was studied in detail in [2], [36] and we expect their results should hold also in the present case.

## Discussion

### MicFFLs role in the regulatory network

The main outcomes of the analyses discussed in this paper are that (i) miRNA-controlled feed-forward loops are able to fine-tune the TF-T ratio to any desired value as a function of the miRNA concentration and (ii) the peculiar topology of the circuit ensures a remarkable stability of this ratio against stochastic fluctuations. These two effects can be traced back to the titrative form of the miRNA-target interplay [16] which in turn induces an indirect TF-T interaction in competition with the standard transcriptional regulation. The additional interaction is controlled by the miRNA concentration which thus fine-tunes the TF-T ratio. The sum of direct and indirect effects results in a stronger TF-T correlation, available for a broader range of miRNA concentration and interaction strengths with respect to any other topology involving the three players (as confirmed by the comparison with the null models we studied). Such peculiar property of micFFLs could be very useful when TF and T must keep fixed concentration ratios, for instance if they must interact with a given stoichiometry. This is for instance the case of (i) TF-T pairs involved in switch-like functions, as those controlling processes of tissue differentiation and cell proliferation, or (ii) TF-TF pairs which cooperate in regulating the same target. Indeed micFFLs involving proximal and distal regulators acting on the same gene are strongly enriched in the human regulatory network [10]. At the same time it is clear that in a generic situation such TF-T linkage should be avoided: the typical outcome of transcriptional regulation is that a small change in the regulator induces a much larger response in the regulated gene. This explains why this motif shows a strong negative enrichment when we reshuffle the transcriptional links. The strong positive enrichment we observe when reshuffling the post-transcriptional side of the network suggests instead that inducing a robust and stable fine-tuning of the TF-T ratio could be one of the most important roles of miRNAs in the regulatory network. In order to elucidate this point we performed two further analyses: a functional enrichment analysis of the micFFL targets and a comparison of the TF-T pairs with the PrePPI database of protein-protein interactions.

### Functional enrichment

We performed a functional analysis of the target gene list corresponding to the FFLs obtained with (i) the JASPAR TF list validated by all 4 miRNA-target databases and (ii) the ENCODE TF list. We used DAVID algorithm [37], [38], a comprehensive set of functional annotation tools, to understand biological meaning behind large lists of genes. We searched for enrichment based on Gene Ontology terms, Kegg metabolic pathways and human deseases. We found for a few categories an impressive enrichment (Bonferroni corrected p-values below 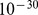). Remarkably enough the two lists of FFLs showed similar enrichment patterns and the most enriched categories turned out to be exactly the expected ones: regulation of transcription, regulation of cell proliferation, positive regulation of cell differentiation, cell cycle and pathways in cancer. We report in Supporting Information Table S1 (for the Jaspar list) and Supporting Information Table S2 (for the Encode list), the complete list of enriched categories with a False Discovery Ratio below 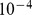.

### MicFFLs with experimentally validated interactions

In order to decrease the number of false positives in the list of putative micFFLs we selected those for which each one of the three regulatory interactions was experimentally validated in at least one experiment. This does not mean that all the three interactions are present in the same biological conditions or that the circuit is effectively active but it is certainly a strong indication in this direction. The list combines information collected from several databases (see details in section Material and Methods). We obtained in this way a list of 499 micFFLs involving 365 distinct TF-T pairs which are reported in Supporting Information Tables S3, S4 and S5. We consider this list as our best candidates for a possible experimental validation of the micFFL properties discussed in the previous sections.

### Comparison with the PrePPI database

We tested the conjecture that micFFLs could have a role in stabilizing the stoichiometric ratio of proteins involved in physical interactions by comparing our list of best candidate micFFLs with the list of protein-protein interactions collected in the PrePPi database [39]. Interactions in the database are validated through an algorithm based on 3 d structure and functional analysis of the polypeptide chain. The algorithm was trained on the interactions of the major databases known till August 2010 and checked through the new interactions noted between august 2010 and august 2011. After training, Zhang's group predicted about 700 new interactions added to the PrePPI database. We found that 30 out of the 499 TF-T pairs were present in the PrePPi database while the expected number was less than one. Assuming a binomial distribution we found a p-value smaller than 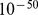. It is clear that we should consider this value with caution, since both PrePPi and our databases contain statistically biased experimentally validated data. However, the gap between the number of expected interactions and those we actually found is so large that it strongly supports our conjecture that micFFLs fine-tune and stabilize the relative concentrations of interacting proteins.

### A prototypical example: The micFFL involving E2F1 and RB1 as targets and a set of miRNAs (miR-106a,miR-106b, miR-17, miR-20a and miR-23b) as master regulators

**Figure 6:**
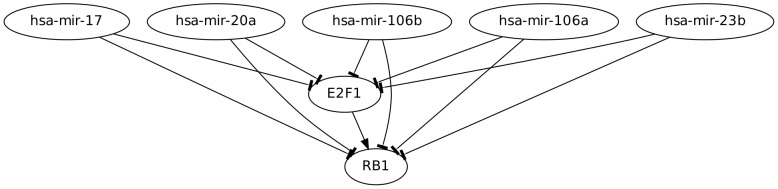
The network of micFFLs involving E2F1 as transcription factor and RB1 as target.

Within the list of candidates with experimentally validated interactions we selected, as an example, the micFFLs involving E2F1 and RB1 as targets and a set of miRNAs (miR-106a, miR-106b miR-17 miR-20a and miR-23b) as master regulators (see Supporting Information Table S4). The network involving these genes is reported in Figure 6. The experimental support for these circuits is very strong (see [10] for the transcriptional regulation and [40] for those involving the miRNAs). E2F1 and RB1 are known to physically interact [33], [41] and are in fact included in the PrePPi database. The E2F1-RB1 system is a well known important switch in the cell cycle. E2F1 belongs to the family of E2F genes, which control the transition from G0/G1 to S phase in the cell (the quiescent phase and the first checkpoint phase respectively). In absence of mitogenic stimulation, E2F-dependent gene expression is inhibited by interaction between E2F and members of the retinoblastoma protein family RB (composed by RB1, RBL1 and RBL2) [41]. When mitogens stimulate cells to divide, RB family members are phosphorilated then reducing their binding to E2F. The thus free-from-binding E2F proteins in turn activate expression of their target genes and trigger cell cycle. In G0 phase almost all cells have E2F1 and RB1 proteins bound in complexes [33], [41]. In this state RB inhibits E2F functions and consequently the cell cycle. It is clear that the stability of the relative concentration of the two genes against stochastic fluctuations is of crucial importance for the correct functioning of this checkpoint. Our analysis suggests that this stability is guaranteed by the five miRNAs listed above and by the peculiar topology of the micFFLs they form with their targets. These micFFLs allow a rapid reaction of RB1 in case of bursts of E2F1 production thus avoiding a dangerous erroneous activation of the E2F1 pathway. The fact that the E2F1-RB1 pair is targeted simultaneously by five miRNAs is likely to reinforce the stabilization function. In our databases there are several other instances of TF-T pairs targeted by more than one miRNA. These are most probably the best candidates for further theoretical and experimental studies.

## Materials and Methods

### Construction of the post-transcriptional side of the regulatory network

As potential targets of miRNAs we selected only transcripts corresponding to protein-coding genes completely annotated in Ensembl 68 [27], for a total of 76722 known transcripts. To define miRNA targets we used four freely available databases, chosen so as to have the widest possible spectrum of different prediction strategies. Three of them, doRiNA [23], microRNA.org [24] and TargetScan [25], use algorithms based on sequence search similarity, possibly considering target site evolutionary conservation. The last one, PITA [26], uses an algorithm based on thermodynamic stability of the RNA-RNA duplex, considering free energy minimization. Integrating the four databases we found a total of 4638441 interactions involving 1581 miRNAs. For each miRNA-T link we annotated how many databases confirm the interaction. Then, out of these interactions, we selected those involving only TFs as targets. We based our analysis on two different TFs databases, JASPAR [28], [29] and ENCODE [10]. We found 34614 miRNA-TF interactions for JASPAR list and 39498 for ENCODE list, involving 127 and 121 TFs respectively.

### Construction of the transcriptional side of the regulatory network

TF-T interactions were obtained with two different strategies depending on the TF database. For the JASPAR TF list we used the Position Frequency Matrix (PFM) information contained in the database [29] and constructed a standard search algorithm for transcription factor binding sites (TFBS) within the target promoter region. Following the same procedure adopted in previous works on the subject [3], [42] we choose 1 kb long promoter regions, from 900 bases before the transcription start site (TSS) to 100 bases after the TSS. We used the scoring function proposed in [29], setting the threshold at 0.7 of the max score. We found in this way a total of 948125 interactions. For the ENCODE TF list we used the ChIP-seq data obtained within the framework of the the ENCODE project [10]. These data were obtained for the 121 TFs over 5 main cell lines. We combined together the results of the different cell lines obtaining a total of 45328 TF-T interactions.

### Identification of micFFLs

We constructed the list of putative micFFL simply combining the interaction links obtained above. We obtained a total of 75933600 and 2426300 micFFLs from JASPAR and ENCODE respectively. The whole list of these micFFLs can be found into the data package from Dryad repository [43]. In order to reduce the number of false positives we then selected only the micFFLs with both miRNA regulatory links confirmed by all the four databases. We obtained in this way 129100 micFFLs in the Jaspar case and 3782 in the ENCODE case (Supplementary Tables S6 and S7).

### Identification of micFFLs with experimentally validated regulatory interactions

The list of micFFLs with experimentally validated regulatory interactions was obtained combining information collected from several databases. For the miRNA→T and the miRNA→TF interactions we used the last versions of miRTarBase V 3.5 (updated November, 2012), miRecords V.3 (updated on November, 2010) and miR2Disease (updated on Jun, 2010). We obtained in this way a list of experimentally validated miRNA-T interactions containing 462 miRNAs, 2280 target genes and a total of 4277 independent interactions in human. For TF→T interactions we used data from ENCODE (which contains a total of 44842 regulatory interactions involving 122 TFs and 10104 target genes) and the last version of Tfact(v.2). Tfact contains genes responsive to transcription factors, according to experimental evidence reported in literature. It reports two datasets: (i) a sign sensitive catalogue that indicates the type (up or down) of TF regulation exerted on its targets and (ii) a signless catalogue that includes all regulatory interactions contained in sign sensitive one plus further interactions without the specific type of regulation. Focusing on human the database contains a total 4299 regulatory interactions involving 276 TFs and 1937 target genes. The total number of non-redundant TF-T regulatory interactions obtained combining the two datasets is 48850 with 335 TFs and 10,828 target genes. Combining the two datasets we obtained a total of 499 micFFLs. Out of them 95 involved a target which is itself a TF and for 7 of them the transcriptional regulation is bidirectional (Supporting Information Table S5). For the remaining 88 (Supporting Information Table S4) only one of the TFs regulates the other and there is no reciprocal interaction. Finally, in the remaining 404 micFFLs the target is not a TF (Supporting Information Table S3).

### Simulations and analytic calculations

Analytical results have been obtained with Mathematica 8.0. Simulations present in SI have been obtained implementing Gillespie's direct algorithm [44].

## Supporting Information

Table S1 Functional enrichments for micFFLs obtained with Jaspar list.
(XLS)

Table S2 Functional enrichments for micFFLs obtained with ENCODE list.
(XLS)

Table S3 List of micFFL where transcription factor targets a gene (not TF).
(XLS)

Table S4 List of micFFL where transcription factor targets another transcription factor.
(XLS)

Table S5 List of circuits where a miRNA regulates two genes and, in turn, each gene is a transcription factor regulating the other gene.
(XLS)

Table S6 List of micFFL obtained from Encode data and sifted through the 4 DB filter.
(CSV)

Table S7 List of micFFL obtained from Jaspar data and sifted through the 4 DB filter.
(CSV)

Supporting Information S1 Details about models.
(PDF)

## References

[1] Martinez NJ, Walhout AJM (2009) The interplay between transcripton factors and microRNAs in genome-scale regulatory networks. Bio Essays 31: 435–445. 10.1002/bies.200800212 PMC3118512 19274664

[2] Alon U (2007) Network motifs: theory and experimental approaches. Nature Rev Genet 8: 450–461. 17510665 10.1038/nrg2102

[3] Re A, Corá D, Taverna D, Caselle M (2009) Genome-wide survey of microRNA-transcription factor feed-forward regulatory circuits in human. Molecular BioSystems 5: 854–867. 19603121 10.1039/b900177h PMC2898627

[4] Shalgi R, Lieber D, Oren M, Pilpel Y (2007) Global and local architecture of the mammalian microRNA-transcription factor regulatory network. PLoS Computational Biology 3 (7) e131. 17630826 10.1371/journal.pcbi.0030131 PMC1914371

[5] Tsang J, Zhu J, van Oudenaarden A (2007) MicroRNA-mediated feedback and feedforward loops are recurrent network motifs in mammals. Mol Cell 26: 753–767. 17560377 10.1016/j.molcel.2007.05.018 PMC2072999

[6] Yu X, lin J, Zack DJ, Mendell JT, Qian J (2008) Analysis of regulatory network topology reveals functionally distinct classes of microRNAs. Nucleic Acids Research 36: 6494–6503. 18927108 10.1093/nar/gkn712 PMC2582613

[7] Hornstein E, Shomron N (2006) Canalization of development by microRNAs. Nature Genetics 38: S20–S24. 16736020 10.1038/ng1803

[8] Osella M, Bosia C, Corá D, Caselle M (2011) The role of incoherent microRNA-mediated feedfor- ward loops in noise buffering. PLoS Computational Biology 7: e1001101. 21423718 10.1371/journal.pcbi.1001101 PMC3053320

[9] Bosia C, Osella M, El Baroudi M, Corá D, Caselle M (2012) Gene autoregulation via intronic microRNAs and its functions. BMC Systems Biology 6: 131. 23050836 10.1186/1752-0509-6-131 PMC3534558

[10] Gerstein M, Kundaje A, Hariharan M, Landt S, Yan K, et al (2012) Architecture of the human regulatory network derived from ENCODE data. Nature 489: 91–100. 22955619 10.1038/nature11245 PMC4154057

[11] Sun J, Gong X, Purow B, Zhao Z (2012) Uncovering MicroRNA and Transcription Factor Mediated Regulatory Networks in Glioblastoma. PLoS Comput Biol 8 (7) e1002488. 22829753 10.1371/journal.pcbi.1002488 PMC3400583

[12] Salmena L, Poliseno L, Tay Y, Kats L, Pandolff PP (2011) A ceRNA Hypothesis: The Rosetta Stone of a Hidden RNA Language? Cell 146: 353–358. 21802130 10.1016/j.cell.2011.07.014 PMC3235919

[13] Sumazin P, Yang X, Chiu HS, Chung WJ, Iyer A, et al (2011) An Extensive MicroRNA-Mediated Network of RNA-RNA Interactions Regulates Established Oncogenic Pathways in Glioblastoma. Cell 147: 370–381. 22000015 10.1016/j.cell.2011.09.041 PMC3214599

[14] Mangan S, Zaslaver A, Alon U (2003) The coherent feedforward loop serves as a sign-sensitive delay element in transcription networks. J Mol Biol 334 (2) 197–204. 14607112 10.1016/j.jmb.2003.09.049

[15] Kalir S, Mangan S, Alon U (2005) A coherent feed-forward loop with a sum input function prolongs agella expression in escherichia coli. Molecular Systems Biology 1: 2005.0006. 16729041 10.1038/msb4100010 PMC1681456

[16] Mukherji S, Ebert M, Zheng G, Tsang J, Sharp P, et al (2011) MicroRNAs can generate thresholds in target gene expression. Nature Genetics 43: 854–859. 21857679 10.1038/ng.905 PMC3163764

[17] Elf J, Paulsson J, Berg O, Ehrenberg M (2003) Near-critical phenomena in intracellular metabolite pools. Biophysical Journal 84: 154–170. 12524272 10.1016/S0006-3495(03)74839-5 PMC1302600

[18] Levine E, Zhang Z, Kuhlman T, Hwa T (2007) Quantitative characteristics of gene regulation by small RNA. PLoS Biology 5 (9) e229. 17713988 10.1371/journal.pbio.0050229 PMC1994261

[19] Mitarai N, Andersson AMC, Krishna S, Semsey S, Sneppen K (2007) Efficient degradation and expression prioritization with small RNAs. Physical Biology 4: 164. 17928655 10.1088/1478-3975/4/3/003

[20] Bosia C, Pagnani A, Zecchina R (2013) Modelling competing endogenous RNA networks. PLoS ONE 8 (6) e66609. 23840508 10.1371/journal.pone.0066609 PMC3694070

[21] Figliuzzi M, De Martino A, Marinari E (2013) MicroRNAs as a selective, post-transcriptional channel of communication between ceRNAs: a steady-state theory. Biophysical Journal 104: 1203–13. 23473503 10.1016/j.bpj.2013.01.012 PMC3870798

[22] Noorbakhsh J, Lang A, Mehta P (2013) Intrinsic Noise of microRNA-Regulated Genes and the ceRNA Hypothesis. PLoS ONE 8 (8) e72676. 23991139 10.1371/journal.pone.0072676 PMC3749174

[23] Anders G, Mackowiak SD, Jens M, Maaskola J, Kuntzagk A, et al (2012) doRiNA: a database of RNA interactions in post-transcriptional regulation. Nucleic Acids Research 40: D180–D186. 22086949 10.1093/nar/gkr1007 PMC3245013

[24] Betel D, Wilson M, Gabow A, Marks DS, Sander C (2008) The microRNA.org resource: targets and expression. Nucleic Acids Research 36: D149–D153. 18158296 10.1093/nar/gkm995 PMC2238905

[25] Lewis B, Shih I, Jones-Rhoades M, Bartel D, Burge C (2003) Prediction of mammalian microRNA targets. Cell 115: 787–798. 14697198 10.1016/s0092-8674(03)01018-3

[26] Kertesz M, Iovino N, Unnerstall U, Gaul U, Segal E (2007) The role of site accessibility in mi- croRNA target recognition. Nature Genetics 39: 1278–1284. 17893677 10.1038/ng2135

[27] Flicek P, Amode MR, Barrell D, Beal K, Brent S, et al (2012) Ensembl 2012. Nucleic Acids Research 40: D84–D90. 22086963 10.1093/nar/gkr991 PMC3245178

[28] Stormo G (2000) DNA binding sites: representation and discovery. Bioinformatics 16: 16–23. 10812473 10.1093/bioinformatics/16.1.16

[29] Wasserman W, Sandelin A (2004) Applied bioinformatics for the identification of regulatory elements. Nature Review Genetics 5: 276–287. 10.1038/nrg1315 15131651

[30] Alon U (2006) An introduction to systems biology: design principles of biological circuits, volume 10. Chapman & Hall/CRC.

[31] Murugan R (2012) Theory on the dynamics of feedforward loops in the transcription factor networks. PLoS ONE 7 (7) e41027. 22911735 10.1371/journal.pone.0041027 PMC3401222

[32] Van Kampen NG (2007) Stochastic processes in physics and chemistry. Elsevier Science & Technology Books, 3 edition.

[33] Alberts (2008) Molecular Biology of the Cell. Garland Science, 5 edition.

[34] Milo R, Jorgensen P, Moran U, Weber G, Springer M (2010) Bionumbers - the database of key numbers in molecular and cell biology. Nucleic acids research 38: D750–D753. 19854939 10.1093/nar/gkp889 PMC2808940

[35] Ala U, Karreth FA, Bosia C, Pagnani A, Taulli R, et al (2013) Integrated transcriptional and competitive endogenous RNA networks are cross-regulated in permissive molecular environments. PNAS 110 (18) 7154–7159. 23536298 10.1073/pnas.1222509110 PMC3645534

[36] Murugan R, Kreiman G (2011) On the minimization of uctuations in the response times of autoregulatory gene networks. Biophysical journal 101: 1297–1306. 21943410 10.1016/j.bpj.2011.08.005 PMC3177052

[37] Huang DW, Sherman BT, Lempicki RA (2009) Systematic and integrative analysis of large gene lists using DAVID bioinformatics resources. Nature Protocols 4: 44–57. 19131956 10.1038/nprot.2008.211

[38] Huang DW, Sherman BT, Lempicki RA (2009) Bioinformatics enrichment tools: paths toward the comprehensive functional analysis of large gene lists. Nucleic Acids Research 37: 1–13. 19033363 10.1093/nar/gkn923 PMC2615629

[39] Zhang QC, Petrey D, Deng L, Qiang L, Shi Y, et al (2012) Structure-based prediction of protein-protein interactions on a genome-wide scale. Nature 490 (7421) 556–60. 23023127 10.1038/nature11503 PMC3482288

[40] Trompeter HI, Abbad H, Iwaniuk KM, Hafner M, Renwick N, et al (2011) MicroRNAs MiR-17, MiR-20a, and MiR-106b Act in Concert to Modulate E2F Activity on Cell Cycle Arrest during Neuronal Lineage Differentiation of USSC. PLoS ONE 6 (1) e16138. 21283765 10.1371/journal.pone.0016138 PMC3024412

[41] Goodrich D (2006) The retinoblastoma tumor-suppressor gene, the exception that proves the rule. Oncogene 25: 5233–5243. 16936742 10.1038/sj.onc.1209616 PMC2799241

[42] Friard O, Re A, Taverna D, De Bortoli M, Corá D (2010) CircuitsDB: a database of mixed mi-croRNA/transcription factor feed-forward regulatory circuits in human and mouse. BMC Bioin-formatics 11: 435. 10.1186/1471-2105-11-435 PMC2936401 20731828

[43] Riba A, Bosia C, Ollino L, El Baroudi M, Caselle M (2014). Data from: A combination of transcriptional and microrna regulation improves the stability of the relative concentrations of target genes. doi:10.5061/dryad.f3h72. 10.1371/journal.pcbi.1003490 PMC3937125 24586138

[44] Gillespie D (1976) A general method for numerically simulating the stochastic time evolution of coupled chemical reactions. Journal of Computational Physics 22: 403–434.

