# Correction: A Combination of Transcriptional and MicroRNA Regulation Improves the Stability of the Relative Concentrations of Target Genes

**DOI:** 10.1371/journal.pcbi.1003582

**Published:** 2014-03-26

**Authors:** The PLOS Computational Biology Staff

The affiliation for the second author is incorrect. Carla Bosia is not affiliated with Department of Physics and INFN, University of Torino, Torino, Italy but with Human Genetics Foundation (HuGeF), Torino, Italy. The correct affiliation is listed as #2 on the PDF but is not present on the HTML version of the paper.
