# Supplementary material for: A Combination of Transcriptional and MicroRNA Regulation Improves the Stability of the Relative Concentrations of Target Genes
Source: PLoS Comput Biol. 2014 Feb 27;10(2):e1003490. doi: 10.1371/journal.pcbi.1003490 (PMC3937125; doi:10.1371/journal.pcbi.1003490)
Supplement: Supporting Information S1 — Details about models. (PDF) [file pcbi.1003490.s008.pdf]

A combination of transcriptional and microRNA regulation improves the stability of the relative concentrations of target genes
Supplementary Information

Andrea Riba, Carla Bosia, Mariama El Baroudi, Laura Ollino, Michele Caselle

Contents

1 Models in detail **2**

2 Master equation **4**

3 Linear noise approximation and simulations: steady state discrepancy **6**

4 Comparison between NM4 and micFFL **7**

5 Comparison with the explicit promoter dynamics **10**

6 Stability analysis **10**

7 Steady state analysis with the logic approximation **12**

1 Models in detail

Gillespie reactions In the following we briefly introduce the reactions involved in the circuits considered in the main text that we used to simulate the models through the implementation of Gillespie's direct algorithm [43]. As discussed in the text, we assume a titrative interaction of the miRNA with both its targets while the interaction between transcription factor (TF) and target (T) is modelled as a Hill function.

Set of reactions for the NM1:

Reaction			*Rate*
$*$	$\to$	m_1	$k^*_{m_1}$
m_1	$\to$	$m_1 + p_1$	$k^*_{p_1} m_1$
p_1	$\to$	$p_1 + m_2$	$k^*_{m_2} \frac{(p_1)^n}{(p_1)^n + h^n}$
m_2	$\to$	$m_2 + p_2$	$k^*_{p_2} m_2$
m_1	$\to$		$\gamma^*_{m_1} m_1$
p_1	$\to$		$\gamma^*_{p_1} p_1$
m_2	$\to$		$\gamma^*_{m_2} m_2$
p_2	$\to$		$\gamma^*_{p_2} p_2$

Picture

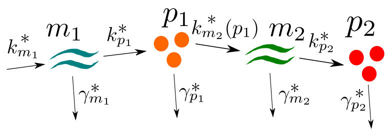

(1)

Set of reaction for the NM2:

Reaction			*Rate*
$*$	$\to$	s	k^*_s
$*$	$\to$	m_1	$k^*_{m_1}$
m_1	$\to$	$m_1 + p_1$	$k^*_{p_1} m_1$
$s + m_1$	$\to$	c_1	$k^{on*}_{c_1} s \cdot m_1$
c_1	$\to$	s	$\alpha \gamma^*_{c_1} c_1$
p_1	$\to$	$p_1 + m_2$	$k^*_{m_2} \frac{(p_1)^n}{(p_1)^n + h^n}$
m_2	$\to$	$m_2 + p_2$	$k^*_{p_2} m_2$
s	$\to$		$\gamma^*_s s$
m_1	$\to$		$\gamma^*_{m_1} m_1$
c_1	$\to$		$(1-\alpha)\gamma^*_{c_1} c_1$
p_1	$\to$		$\gamma^*_{p_1} p_1$
m_2	$\to$		$\gamma^*_{m_2} m_2$
p_2	$\to$		$\gamma^*_{p_2} p_2$

Picture

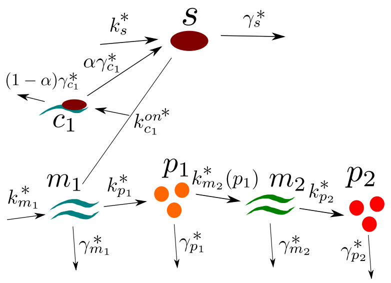

(2)

Set of reaction for the NM3:

Reaction			*Rate*
$*$	$\rightarrow$	s	k_s^*
$*$	$\rightarrow$	m_1	$k_{m_1}^*$
m_1	$\rightarrow$	m_1+p_1	$k_{p_1}^* m_1$
p_1	$\rightarrow$	p_1+m_2	$k_{m_2}^* \frac{(p_1)^n}{(p_1)^n+h^n}$
m_2	$\rightarrow$	m_2+p_2	$k_{p_2}^* m_2$
$s+m_2$	$\rightarrow$	c_2	$k_{c_2}^{on*} s \cdot m_2$
c_2	$\rightarrow$	s	$\alpha\gamma_{c_2}^* c_2$
s	$\rightarrow$		$\gamma_s^* s$
m_1	$\rightarrow$		$\gamma_{m_1}^* m_1$
p_1	$\rightarrow$		$\gamma_{p_1}^* p_1$
m_2	$\rightarrow$		$\gamma_{m_2}^* m_2$
c_2	$\rightarrow$		$(1-\alpha)\gamma_{c_2}^* c_2$
p_2	$\rightarrow$		$\gamma_{p_2}^* p_2$

Picture

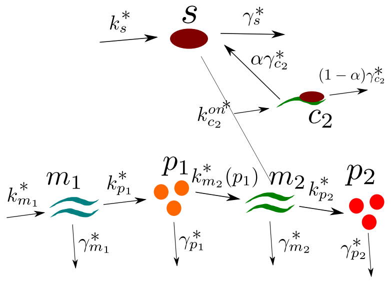

(3)

Set of reaction for the maimed NM4:

Reaction			*Rate*
$*$	$\rightarrow$	s	k_s^*
$*$	$\rightarrow$	m_1	$k_{m_1}^*$
m_1	$\rightarrow$	m_1+p_1	$k_{p_1}^* m_1$
$s+m_1$	$\rightarrow$	c_1	$k_{c_1}^{on*} s \cdot m_1$
c_1	$\rightarrow$	s	$\alpha\gamma_{c_1}^* c_1$
$*$	$\rightarrow$	m_2	k_{m_2}
m_2	$\rightarrow$	m_2+p_2	$k_{p_2}^* m_2$
$s+m_2$	$\rightarrow$	c_2	$k_{c_2}^{on*} s \cdot m_2$
c_2	$\rightarrow$	s	$\alpha\gamma_{c_2}^* c_2$
s	$\rightarrow$		$\gamma_s^* s$
m_1	$\rightarrow$		$\gamma_{m_1}^* m_1$
c_1	$\rightarrow$		$(1-\alpha)\gamma_{c_1}^* c_1$
p_1	$\rightarrow$		$\gamma_{p_1}^* p_1$
m_2	$\rightarrow$		$\gamma_{m_2}^* m_2$
c_2	$\rightarrow$		$(1-\alpha)\gamma_{c_2}^* c_2$
p_2	$\rightarrow$		$\gamma_{p_2}^* p_2$

Picture

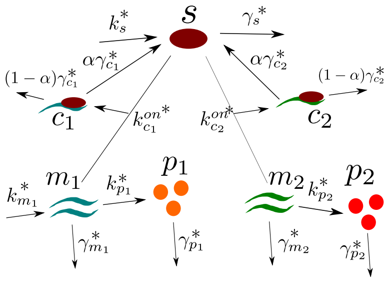

(4)

Set of reaction for the NM5:

Reaction			Rate	Picture
$*$	$\rightarrow$	s_1	k_s^*	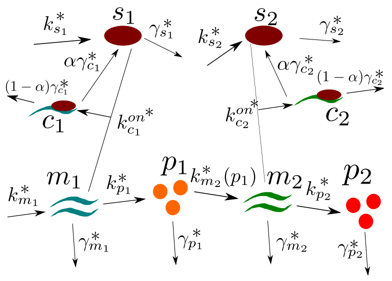
$*$	$\rightarrow$	s_2	k_s^*	
$*$	$\rightarrow$	m_1	$k_{m_1}^*$	
$s_1 + m_1$	$\rightarrow$	c_1	$k_{c_1}^{on*} s_1 \cdot m_1$	
c_1	$\rightarrow$	s_1	$\alpha \gamma_{c_1}^* c_1$	
m_1	$\rightarrow$	$m_1 + p_1$	$k_{p_1}^* m_1$	
p_1	$\rightarrow$	$p_1 + m_2$	$k_{m_2}^* \frac{(p_1)^n}{(p_1)^n + h^n}$	
m_2	$\rightarrow$	$m_2 + p_2$	$k_{p_2}^* m_2$	
$s_2 + m_2$	$\rightarrow$	c_2	$k_{c_2}^{on*} s_2 \cdot m_2$	
c_2	$\rightarrow$	s_2	$\alpha \gamma_{c_2}^* c_2$	
s_1	$\rightarrow$		$\gamma_s^* s_1$	
s_2	$\rightarrow$		$\gamma_s^* s_2$	
m_1	$\rightarrow$		$\gamma_{m_1}^* m_1$	
c_1	$\rightarrow$		$(1-\alpha)\gamma_{c_1}^* c_1$	
p_1	$\rightarrow$		$\gamma_{p_1}^* p_1$	
m_2	$\rightarrow$		$\gamma_{m_2}^* m_2$	
c_2	$\rightarrow$		$(1-\alpha)\gamma_{c_2}^* c_2$	
p_2	$\rightarrow$		$\gamma_{p_2}^* p_2$	

(5)

Set of reaction for the micFFL:

Reaction			Rate	Picture
$*$	$\rightarrow$	s	k_s^*	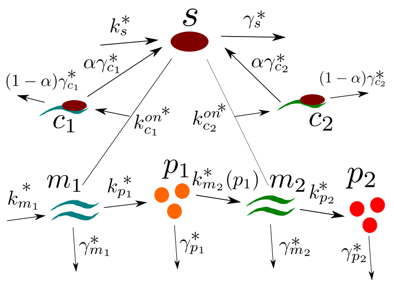
$*$	$\rightarrow$	m_1	$k_{m_1}^*$	
$s + m_1$	$\rightarrow$	c_1	$k_{c_1}^{on*} s \cdot m_1$	
c_1	$\rightarrow$	s	$\alpha \gamma_{c_1}^* c_1$	
m_1	$\rightarrow$	$m_1 + p_1$	$k_{p_1}^* m_1$	
p_1	$\rightarrow$	$p_1 + m_2$	$k_{m_2}^* \frac{(p_1)^n}{(p_1)^n + h^n}$	
m_2	$\rightarrow$	$m_2 + p_2$	$k_{p_2}^* m_2$	
$s + m_2$	$\rightarrow$	c_2	$k_{c_2}^{on*} s \cdot m_2$	
c_2	$\rightarrow$	s	$\alpha \gamma_{c_2}^* c_2$	
s	$\rightarrow$		$\gamma_s^* s$	
m_1	$\rightarrow$		$\gamma_{m_1}^* m_1$	
c_1	$\rightarrow$		$(1-\alpha)\gamma_{c_1}^* c_1$	
p_1	$\rightarrow$		$\gamma_{p_1}^* p_1$	
m_2	$\rightarrow$		$\gamma_{m_2}^* m_2$	
c_2	$\rightarrow$		$(1-\alpha)\gamma_{c_2}^* c_2$	
p_2	$\rightarrow$		$\gamma_{p_2}^* p_2$	

(6)

2 Master equation

In the following we report the master equations for the various circuits analyzed, with $\{s \rightarrow n_1, m_1 \rightarrow n_2, c_1 \rightarrow n_3, p_1 \rightarrow n_4, m_2 \rightarrow n_5, c_2 \rightarrow n_6, p_2 \rightarrow n_7\}$ and the step operator $\mathcal{E}_j^k = \sum_{l=0}^{\infty} \frac{k^l}{l!} \frac{\partial^l}{\partial n_j^l}$ as defined in [32]. The rates are rescales as in the main text.

NM1

$$
\begin{aligned}
\frac{\partial P(\{n_i\},\tau)}{\partial \tau} &= \big\{ k_{m_1}(\mathcal{E}_2^{-1}-1) + \gamma_{m_1}(\mathcal{E}_2^{1}-1)n_2 k_{p_1} n_2 (\mathcal{E}_4^{-1}-1) + \\
&+\gamma_{p_1}(\mathcal{E}_4^{1}-1)n_4 + k_{m_2}\textstyle\sum_n C_n n_4^n (\mathcal{E}_5^{-1}-1) + \\
&+\gamma_{m_2}(\mathcal{E}_5^{1}-1)n_5 + k_{p_2} n_5 (\mathcal{E}_7^{-1}-1) + \\
&+(\mathcal{E}_7^{1}-1)n_7 \big\} P(\{n_i\},t) \; ;
\end{aligned}
\tag{7}
$$

NM3

$$
\begin{aligned}
\frac{\partial P(\{n_i\},\tau)}{\partial \tau} &= \big\{ k_s(\mathcal{E}_1^{-1}-1) + \gamma_s(\mathcal{E}_1^{1}-1)n_1 + k_{m_1}(\mathcal{E}_2^{-1}-1) + \\
&+\gamma_{m_1}(\mathcal{E}_2^{1}-1)n_2 + k_{p_1} n_2 (\mathcal{E}_4^{-1}-1) + \gamma_{p_1}(\mathcal{E}_4^{1}-1)n_4 + \\
&+k_{m_2}\textstyle\sum_n C_n n_4^n (\mathcal{E}_5^{-1}-1) + \gamma_{m_2}(\mathcal{E}_5^{1}-1)n_5 + \\
&+k_{c_2}^{on}(\mathcal{E}_1^{1}\mathcal{E}_5^{1}\mathcal{E}_6^{-1}-1)n_1 n_5 + \alpha\gamma_{c_2}(\mathcal{E}_1^{-1}\mathcal{E}_6^{1}-1)n_6 + \\
&+k_{p_2} n_5 (\mathcal{E}_7^{-1}-1) + (\mathcal{E}_7^{1}-1)n_7 + (1-\alpha)\gamma_{c_2}(\mathcal{E}_6^{1}-1)n_6 \big\} P(\{n_i\},t) \; ;
\end{aligned}
\tag{8}
$$

NM4

$$
\begin{aligned}
\frac{\partial P(\{n_i\},\tau)}{\partial \tau} &= \big\{ k_s(\mathcal{E}_1^{-1}-1) + \gamma_s(\mathcal{E}_1^{1}-1)n_1 + k_{m!}(\mathcal{E}_2^{-1}-1) + \\
&+\gamma_{m_1}(\mathcal{E}_2^{1}-1)n_2 + k_{c_1}^{on}(\mathcal{E}_1^{1}\mathcal{E}_2^{1}\mathcal{E}_3^{-1}-1)n_1 n_2 + \\
&+\alpha\gamma_{c_1}(\mathcal{E}_1^{-1}\mathcal{E}_3^{1}-1)n_3 + k_{p_1} n_2 (\mathcal{E}_4^{-1}-1) + \gamma_{p_1}(\mathcal{E}_4^{1}-1)n_4 + \\
&+k_{m_2}(\mathcal{E}_5^{-1}-1) + \gamma_{m_2}(\mathcal{E}_5^{1}-1)n_5 + \\
&+k_{c_2}^{on}(\mathcal{E}_1^{1}\mathcal{E}_5^{1}\mathcal{E}_6^{-1}-1)n_1 n_5 + \gamma_{c_2}(\mathcal{E}_1^{-1}\mathcal{E}_6^{1}-1)n_6 + \\
&+k_{p_2} n_5 (\mathcal{E}_7^{-1}-1) + (\mathcal{E}_7^{1}-1)n_7 + (1-\alpha)\gamma_{c_1}(\mathcal{E}_3^{1}-1)n_3 + \\
&+(1-\alpha)\gamma_{c_2}(\mathcal{E}_6^{1}-1)n_6 \big\} P(\{n_i\},t) \; ;
\end{aligned}
\tag{9}
$$

NM5

$$
\begin{aligned}
\frac{\partial P(\{n_i\},\tau)}{\partial \tau} &= \big\{ k_{s_1}(\mathcal{E}_1^{-1}-1) + \gamma_{s_1}(\mathcal{E}_1^{1}-1)n_1 + \\
&+k_{s_2}(\mathcal{E}_8^{-1}-1) + \gamma_{s_2}(\mathcal{E}_8^{1}-1)n_8 + k_{m_1}(\mathcal{E}_2^{-1}-1) + \\
&+\gamma_{m_1}(\mathcal{E}_2^{1}-1)n_2 + k_{c_1}^{on}(\mathcal{E}_1^{1}\mathcal{E}_2^{1}\mathcal{E}_3^{-1}-1)n_1 n_2 + \\
&+\alpha\gamma_{c_1}(\mathcal{E}_1^{-1}\mathcal{E}_3^{1}-1)n_3 + k_{p_1} n_2 (\mathcal{E}_4^{-1}-1) + \gamma_{p_1}(\mathcal{E}_4^{1}-1)n_4 + \\
&+k_{m_2}\textstyle\sum_n C_n n_4^n (\mathcal{E}_5^{-1}-1) + \gamma_{m_2}(\mathcal{E}_5^{1}-1)n_5 + \\
&+k_{c_2}^{on}(\mathcal{E}_8^{1}\mathcal{E}_5^{1}\mathcal{E}_6^{-1}-1)n_1 n_5 + \alpha\gamma_{c_2}(\mathcal{E}_8^{-1}\mathcal{E}_6^{1}-1)n_6 + \\
&+k_{p_2} n_5 (\mathcal{E}_7^{-1}-1) + (\mathcal{E}_7^{1}-1)n_7 + (1-\alpha)\gamma_{c_2}(\mathcal{E}_3^{1}-1)n_3 + \\
&+(1-\alpha)\gamma_{c_2}(\mathcal{E}_6^{1}-1)n_6 \big\} P(\{n_i\},t) \; ;
\end{aligned}
\tag{10}
$$

micFFL

$$
\begin{aligned}
\frac{\partial P(\{n_i\},\tau)}{\partial \tau} &= \big\{ k_s(\mathcal{E}_1^{-1}-1) + \gamma_s(\mathcal{E}_1^{1}-1)n_1 + k_{m_1}(\mathcal{E}_2^{-1}-1) + \\
&+\gamma_{m_1}(\mathcal{E}_2^{1}-1)n_2 + k_{c_1}^{on}(\mathcal{E}_1^{1}\mathcal{E}_2^{1}\mathcal{E}_3^{-1}-1)n_1 n_2 + \\
&+\alpha\gamma_{c_1}(\mathcal{E}_1^{-1}\mathcal{E}_3^{1}-1)n_3 + k_{p_1} n_2 (\mathcal{E}_4^{-1}-1) + \gamma_{p_1}(\mathcal{E}_4^{1}-1)n_4 + \\
&+k_{m_2}\textstyle\sum_n C_n n_4^n (\mathcal{E}_5^{-1}-1) + \gamma_{m_2}(\mathcal{E}_5^{1}-1)n_5 + \\
&+k_{c_2}^{on}(\mathcal{E}_1^{1}\mathcal{E}_5^{1}\mathcal{E}_6^{-1}-1)n_1 n_5 + \alpha\gamma_{c_2}(\mathcal{E}_1^{-1}\mathcal{E}_6^{1}-1)n_6 + \\
&+k_{p_2} n_5 (\mathcal{E}_7^{-1}-1) + (\mathcal{E}_7^{1}-1)n_7 + (1-\alpha)\gamma_{c_1}(\mathcal{E}_3^{1}-1)n_3 + \\
&+(1-\alpha)\gamma_{c_2}(\mathcal{E}_6^{1}-1)n_6 \big\} P(\{n_i\},t) \; .
\end{aligned}
\tag{11}
$$

From each master equation we obtained the first two moments of the various distributions through the linear noise approximation (LNA) [32]. Briefly, given R reactions and N molecular species, a general master equation takes the form

$$
\frac{d\,P(\vec{n},t)}{dt} = \Omega \sum_{i=1}^{R} \left(\prod_{j=1}^{N} \mathcal{E}_j^{-v_{ij}} - 1 \right) f_i(\vec{n}\,\Omega^{-1},\Omega) P(\vec{n},t) \tag{12}
$$

where Ω is the volume of the system, v_{ij} is a matrix with dimensions $\#\,reactions \cdot \#\,species$ (in which each element is equal to the concentration change of species j due to reaction i) and $\mathcal{E}$ is the step-operator.

With the following change of variables

$$\vec{n} = \Omega\,\vec{x} + \Omega^{\frac{1}{2}}\vec{\xi} \tag{13}$$

and after its substitution in the master equation, we obtain

$$\frac{\partial\,\Pi}{\partial t} - \Omega^{\frac{1}{2}}\sum_{i=1}^{N}\frac{d\,x_i}{dt}\frac{\partial\Pi}{\partial\xi_i} = \Omega\sum_{i=1}^{R}\left(\prod_{j=1}^{N}\sum_{l=0}^{\infty}\frac{(-v_{ij})^l}{l!}\Omega^{-\frac{l}{2}}\frac{\partial^l}{\partial\xi_i^l} - 1\right) f_i(\vec{x}+\Omega^{-\frac{1}{2}}\vec{\xi})\Pi(\vec{\xi},t)\ . \tag{14}$$

From the highest order ($\Omega^{\frac{1}{2}}$) we obtain the equations for the mean concentrations per unit of volume. From the order Ω^0 and under the assumption that Ω is large enough (so that smaller orders are negligible) we obtain the equations for the fluctuations. Expanding the functions f_i in power of ξ we have to consider only the 0th-order:

$$\frac{\partial\Pi}{\partial t} = -\sum_{j=1}^{N}\left(\sum_{i=1}^{R} v_{ij}\frac{\partial f_i}{\partial\xi_k}\right)\frac{\partial}{\partial\xi_j}(\xi_k\Pi) + \sum_{j,k=1}^{N}\left(\sum_{i=1}^{R} f_i(\vec{x})v_{ij}v_{ik}\right)\frac{\partial}{\partial\xi_j\partial\xi_k}\Pi \tag{15}$$

Then, defining the two matrices

$$\vec{A}_{jk} = \sum_{i=1}^{R} v_{ij}\frac{\partial f_i}{\partial\xi_k} \quad \vec{B}_{jk} = \sum_{i=1}^{R} f_i(\vec{x})v_{ij}v_{ik} \tag{16}$$

and using the multivariate Fokker-Planck equation's solution for the first two moments, we obtain the following relationships:

$$\partial_t < \vec{\xi} >= \vec{A} < \vec{\xi} > \quad \text{with} \ < \vec{\xi} >= (<\xi_1>, \ldots, <\xi_N>)^t\ ; \tag{17}$$

$$\partial_t\Xi = \vec{A}\Xi + \Xi\vec{A}^t + \vec{B} \quad \text{with}\ \Xi_{ij} =< \xi_i\xi_j >\ . \tag{18}$$

From these equations it is straightforward to calculate the approximated correlations $r_{x,y}$ and variances σ_x for each concentration. In supplementary figure 1 we show the comparison between simulations and approximated analytical results.

3 Linear noise approximation and simulations: steady state discrepancy

The main problem of the above analysis is that the LNA does not properly take into account the correlation between chemical species in the evaluation of steady state concentrations. Indeed, if we solve the generating function for the first moments for mRNA and protein we obtain:

$$\begin{aligned} &< m_1 >= \frac{k_{m_1} - k_{c_1}^{on} < s\,m_1 >}{\gamma_{m_1}} \\ &< p_1 >= \frac{k_{p_1}}{\gamma_{p_1}} < m_1 > \\ &< m_2 >= \frac{k_{m_2}\frac{(<p_1>)^n}{(<p_1>)^n + h^n} - k_{c_2}^{on} < s\,m_2 >}{\gamma_{m_2}} \\ &< p_2 >= k_{p_2} < m_2 > \end{aligned} \tag{19}$$

The LNA expectation for the mixed moments is $< xy >=< x >< y >$ while from the Master equation we find

$$< xy >=< x >< y > + \sigma_x\sigma_y r_{xy} = (1 + \eta_x\eta_y r_{xy}) < x >< y > \tag{20}$$

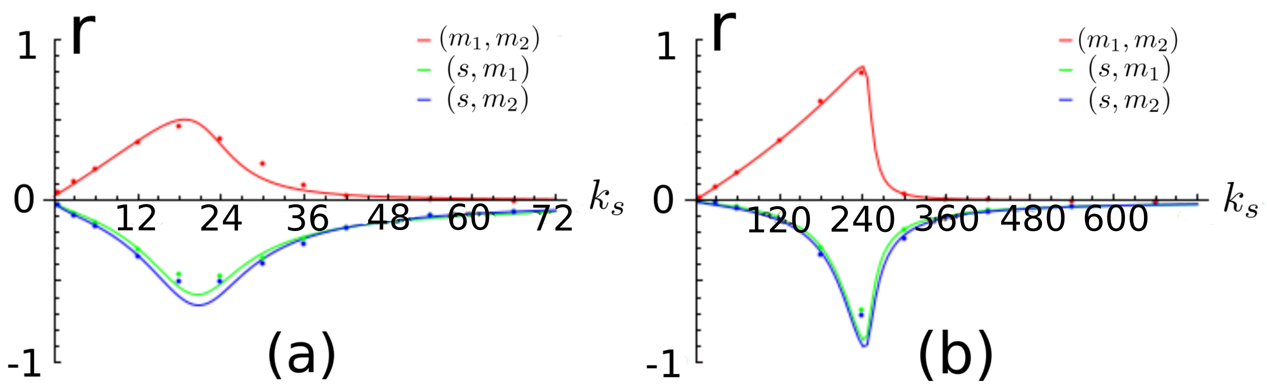

Figure 1. Examples of linear noise approximation and gillespie comparison, measuring correlations r_{m_1,m_2}, r_{s,m_1} and r_{s,m_2},,among s, m_1 and m_2 in the micFFL as a function of the miRNA production rate k_s. Points correspond to stochastic simulations and continuous lines to LNA solutions. **(a)** and **(b)** correspond to two different sets of parameters. **(a)** Parameters:
$k_{m_1} = 23.5$, $k_{m_2} = 41$, $k_{c_1}^{on} = k_{c_2}^{on} = 1.5$, $k_{p_1} = k_{p_2} = 117$, $\gamma_{m_1} = \gamma_{m_2} = 2$, $\gamma_s = \gamma_{p_1} = 1$, $\alpha\gamma_{c_1} = \alpha\gamma_{c_2} = 1.5$, $(1-\alpha)\gamma_{c_1} = (1-\alpha)\gamma_{c_2} = 1$, $h = 200$, $n = 1$ and k_s varies. **(b)** Parameters:
$k_{m_1} = 235$, $k_{m_2} = 410$, $k_{c_1}^{on} = k_{c_2}^{on} = 1.5$, $k_{p_1} = k_{p_2} = 117$, $\gamma_{m_1} = \gamma_{m_2} = 2$, $\gamma_s = \gamma_{p_1} = 1$, $\alpha\gamma_{c_1} = \alpha\gamma_{c_2} = 1.5$, $(1-\alpha)\gamma_{c_1} = (1-\alpha)\gamma_{c_2} = 1$, $h = 200$, $n = 1$ and k_s is variable.

where ηs are measures of noise. Using equation (20) we can rewrite the steady state values corrected with a correlation term

$$\begin{aligned} &< m_1 >= \frac{k_{m_1}}{\gamma_{m_1} + k_{c_1}^{on}(1+\eta_s\eta_{m_1} r_{(s,m_1)}) < s >} \\ &< p_1 >= \frac{k_{p_1}}{\gamma_{p_1}} < m_1 > \\ &< m_2 >= \frac{k_{m_2}\frac{(<p_1>)^n}{(<p_1>)^n + h^n}}{\gamma_{m_2} + k_{c_2}^{on}(1+\eta_s\eta_{m_2} r_{(s,m_2)}) < s >} \\ &< p_2 >= k_{p_2} < m_2 > \end{aligned} \tag{21}$$

As it is possible to notice in Figure 2, these corrections can well explain the discrepancy occurring between simulations and approximated first order differential equations. Notice that the highest values of the correlation are obtained in the parameter region in which concentrations of microRNA and messengers are similar.

4 Comparison between NM4 and micFFL

To better investigate the increase in correlation due to the transcriptional link, we compare NM4 and micFFL (i.e. the two circuits which show the best results in terms of correlation between TF and T). We fix mRNA, protein, microRNA and complexes amounts and then evaluate the ratio between p_1 and p_2 correlations in NM4 and micFFL. The constraint is thus the following:

$$k_{m_2}^{NM4} = k_{m_2}^{micFFL} \frac{p_2^n}{p_2^n + h^n} \ . \tag{22}$$

Thanks to this constraint it is possible to compare directly the gain of correlation due to micFFL with respect to NM4. Our results are shown in Figure 3. The circuit with the transcriptional link between TF and T proteins always reaches higher values of correlation.

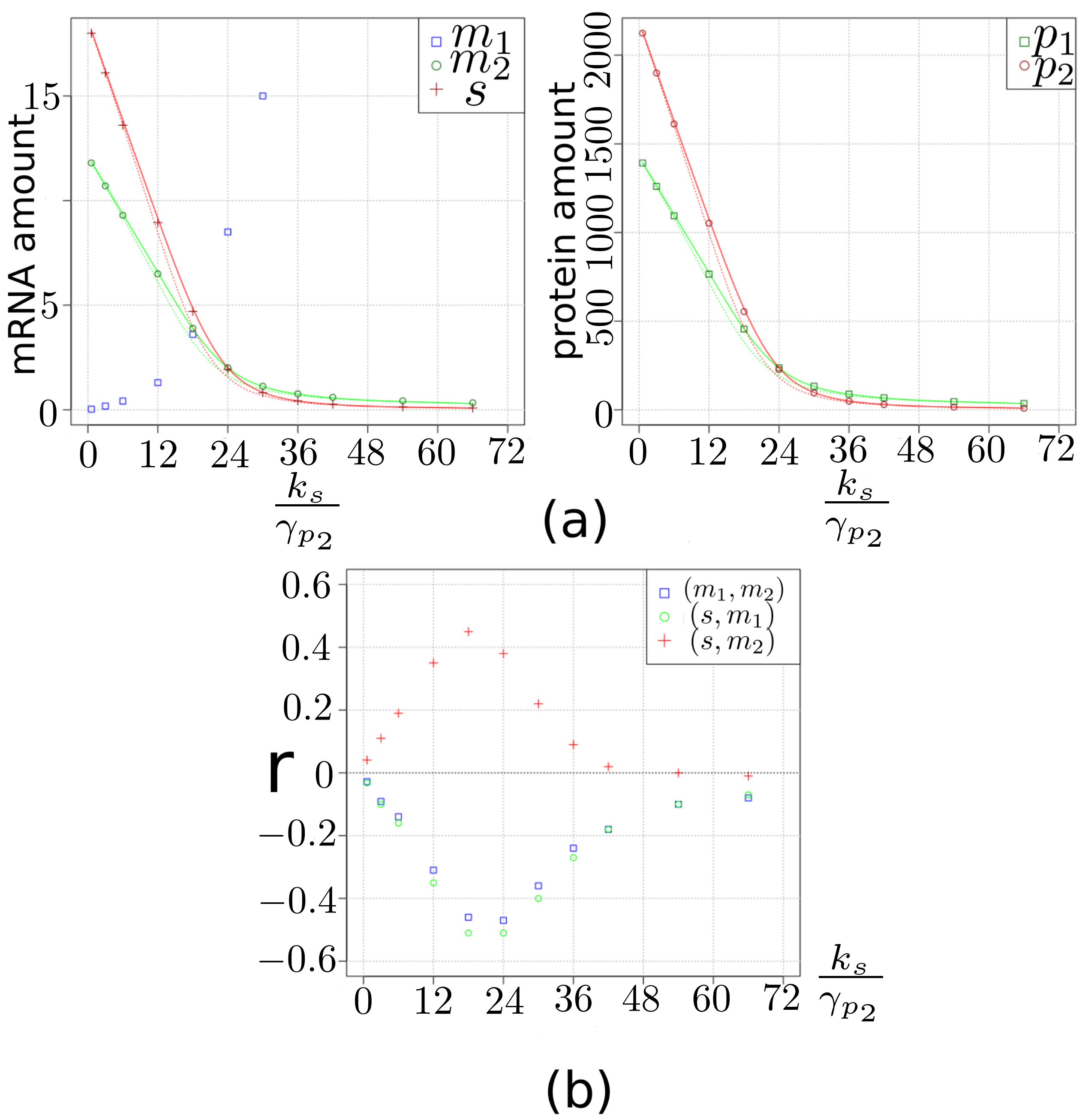

Figure 2. **(a)** Comparison among steady state concentrations. The concentrations of the involved species are plotted against the miRNA transcription rate k_s. Points are simulated data, dotted lines are values expected from the system of first order differetial equations and continuous lines are values expected from the correction of the master equation. **(b)** Correlations between microRNA and messengers. The highest correlated region is located where concentrations of microRNA and messengers are similar. Parameters: $k_{m_1} = 23.5$, $k_{m_2} = 41$, $k_{c_1}^{on} = k_{c_2}^{on} = 1.5$, $k_{p_1} = k_{p_2} = 117$, $\gamma_{m_1} = \gamma_{m_2} = 2$, $\gamma_s = \gamma_{p_1} = 1$, $\alpha\gamma_{c_1} = \alpha\gamma_{c_2} = 1.5$, $(1-\alpha)\gamma_{c_1} = (1-\alpha)\gamma_{c_2} = 1$, $h = 200$, $n = 1$ and k_s is variable.

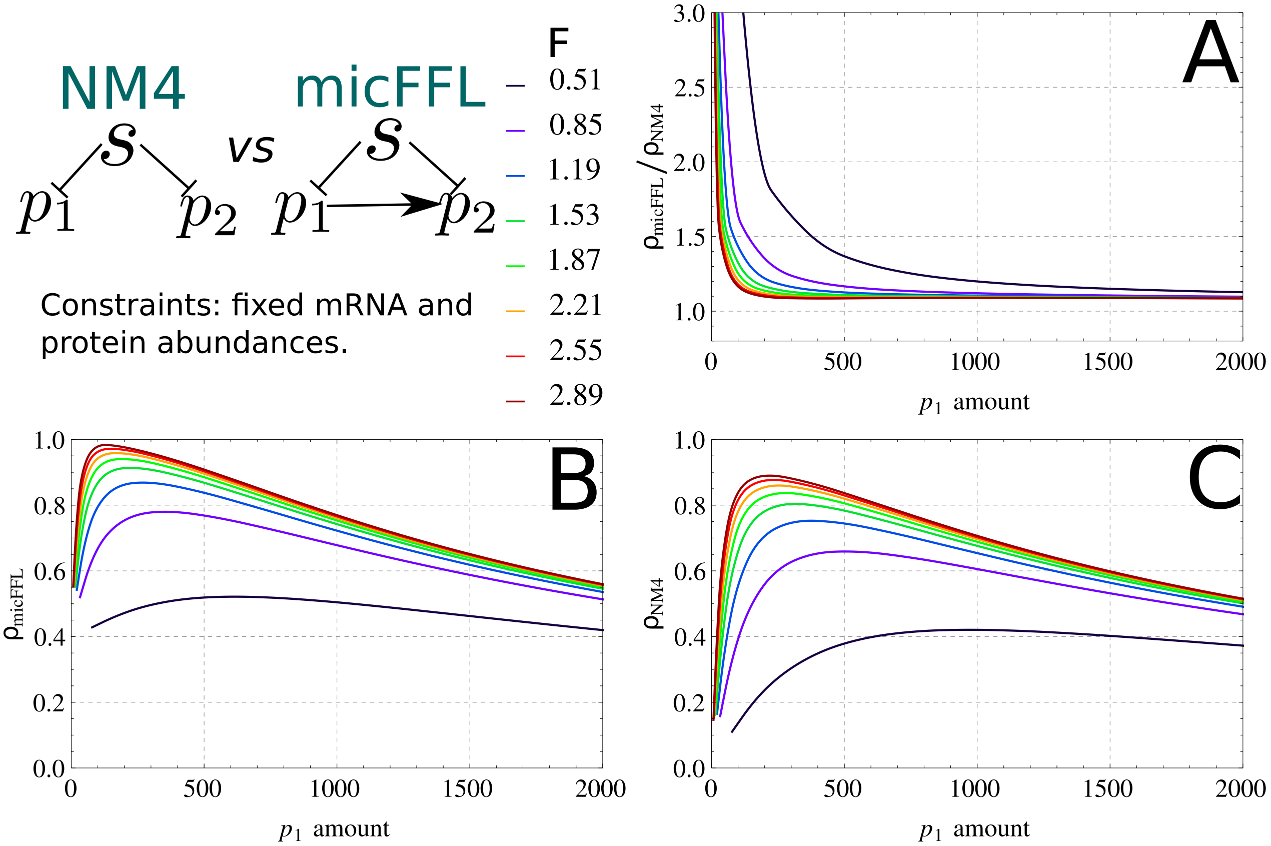

Figure 3. Correlation analysis of NM4 and micFFL fixing the amount of all the molecular species. Correlation values are calculated between p_1 and p_2. **A** Ratio between correlation of micFFL and NM4. Each curve corresponds to different values of the interaction strength F. Varying the amount of TF, micFFL keeps correlation always higher than NM4. **B** Correlation in micFFL varying p_1. **C** Correlation in NM4 varying p_1.

5 Comparison with the explicit promoter dynamics

In all the models used we assumed that the promoter dynamics is much faster than the other reactions. In order to test if such common approximation does not affect our results we substitute the reaction for the target mRNA production with three reactions describing the promoter interaction with the transcription factor (see Table below).

Reaction			*Rate*	*Picture*
p_1	$\rightarrow$	$p_1 + m_2$	$k_{m_2}\frac{p_1}{p_1+h}$	
	$\Downarrow$			
$p_1 + Prom_{off}$	$\rightarrow$	$Prom_{on}$	$k_{on}^{prom} p_1$	
$Prom_{on}$	$\rightarrow$	$m_2 + Prom_{on}$	k_{m_2}	
$Prom_{on}$	$\rightarrow$	$Prom_{off} + p_1$	k_{off}^{prom}	

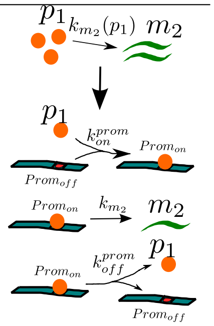

(23)

From the reactions in Table 23 it is possible to calculate the probability of having the TF bound to the promoter. The activated promoter (p_{bound}) can then produce mRNA with a fixed rate:

$$\begin{aligned} p_{bound} &= \frac{k_{on}^{prom} p_1}{k_{off}^{prom} + k_{on}^{prom} p_1} \\ k_{m_2}(p_1) &= k_{m_2}\frac{k_{on}^{prom} p_1}{k_{off}^{prom} + k_{on}^{prom} p_1} \end{aligned} \tag{24}$$

Comparing the two models (without and with the explicit promoter), we find the following mapping:

$$k_{m_2}\frac{p_1^n}{h^n + p_1^n} = k_{m_2}\frac{k_{on}^{prom} p_1}{k_{off}^{prom} + k_{on}^{prom} p_1} \Rightarrow \qquad n = 1, \quad h = \frac{k_{off}^{prom}}{k_{on}^{prom}}. \tag{25}$$

Figure 4 shows that for fast promoter dynamics there is a very good agreement between the two models in terms of mean number of molecules (Figure 4A and B), noise (Figure 4C) and correlation coefficient (Figure 4D). Each curve is the mean over 1000 Gillespie's trajectories.

6 Stability analysis

In this section we accomplish a brief stability analysis of the micFFL circuit. From the definition

$$\frac{d}{dt}\overrightarrow{x} = \overrightarrow{F}(\overrightarrow{x}) \tag{26}$$

we expand the vectors for the micFFL case, so that

$$\begin{cases} \frac{ds}{d\tau} = k_s - \gamma_s s - k_{c_1}^{on} s\, m_1 + \alpha\gamma_{c_1} c_1 - k_{c_2}^{on} s\, m_2 + \alpha\gamma_{c_2} c_2 \\ \frac{dm_1}{d\tau} = k_{m_1} - \gamma_{m_1} m_1 - k_{c_1}^{on} s\, m_1 \\ \frac{dc_1}{d\tau} = k_{c_1}^{on} s\, m_1 - \gamma_{c_1} c_1 \\ \frac{dp_1}{d\tau} = k_{p_1} m_1 - \gamma_{p_1} p_1 \\ \frac{dm_2}{d\tau} = k_{m_2}\frac{p_1^n}{p_1^n + h^n} - \gamma_{m_2} m_2 - k_{c_2}^{on} s\, m_2 \\ \frac{dc_2}{d\tau} = k_{c_2}^{on} s\, m_2 - \gamma_{c_2} c_2 \\ \frac{dp_2}{d\tau} = k_{p_2} m_2 - p_2 \end{cases} \tag{27}$$

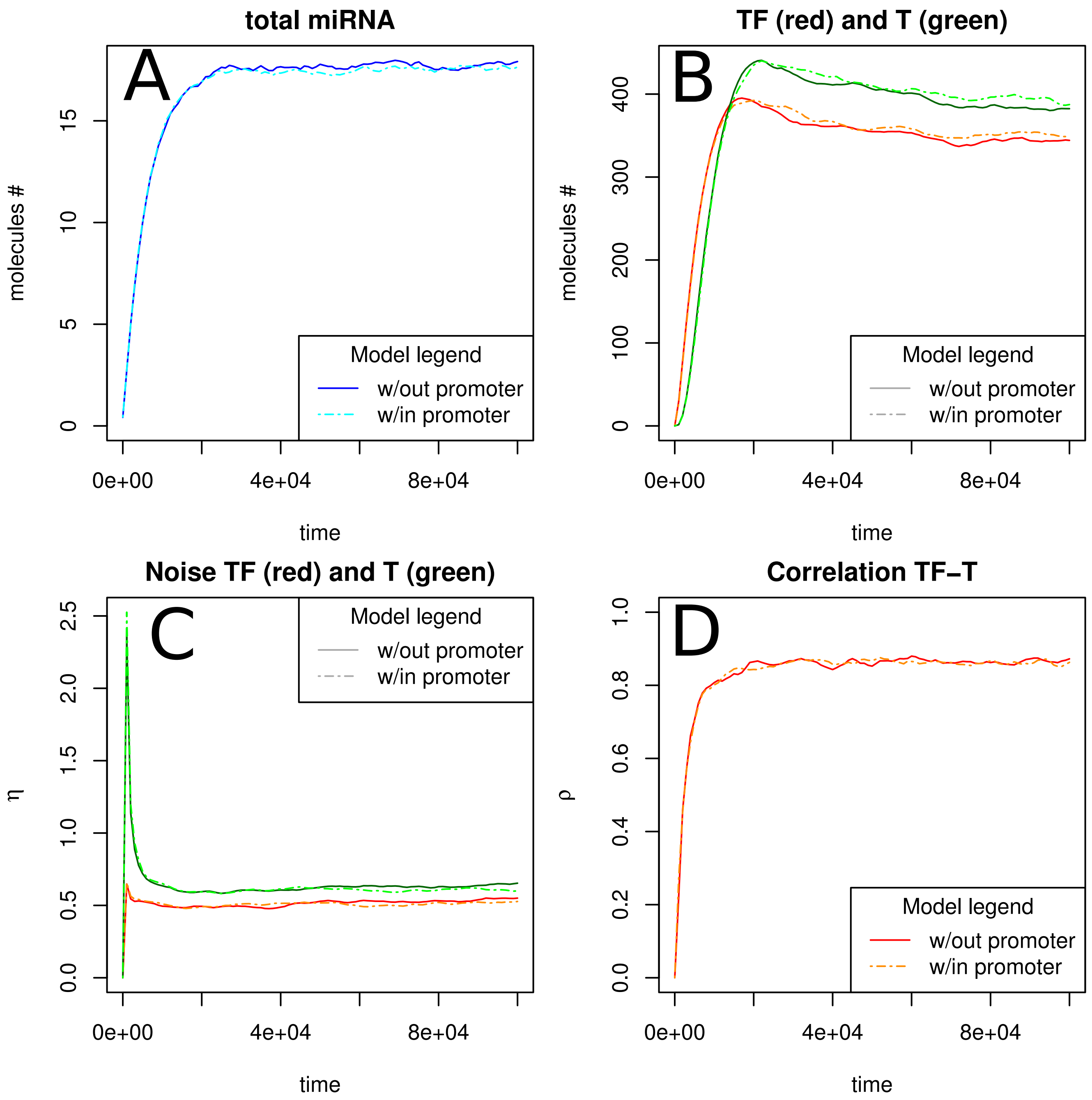

Figure 4. Comparison between Gillespie simulations with promoter model or with Michelis-Menten function. Parameters: $k_{m_1} = 23.5$, $k_{m_2} = 41$, $k_{c_1}^{on} = k_{c_2}^{on} = 1.5$, $k_{p_1} = k_{p_2} = 117$, $\gamma_{m_1} = \gamma_{m_2} = 2$, $\gamma_s = \gamma_{p_1} = 1$, $\alpha\gamma_{c_1} = \alpha\gamma_{c_2} = 1.5$, $(1-\alpha)\gamma_{c_1} = (1-\alpha)\gamma_{c_2} = 1$, $k_{on}^{prom} = 6$, $k_{off}^{prom} = 1000$, $h = 200$, $n = 1$ and k_s is variable.

Depending on the sign of $\nabla \cdot \overrightarrow{F}$ the system could be dissipative ($\nabla \cdot \overrightarrow{F} \leq 0$) or conservative ($\Leftrightarrow \nabla \cdot \overrightarrow{F} = 0$). In our particular case the system is dissipative:

$$\nabla \cdot \overrightarrow{F}(\overrightarrow{x}) = -\gamma_{c_1} - \gamma_{c_2} - \gamma_s - \gamma_{m_1} - \gamma_{m_2} - \gamma_{p_1} - (k^{on}_{c_1} + k^{on}_{c_2})s - k^{on}_{c_1} m_1 - k^{on}_{c_2} m_2 < 0\,. \qquad (28)$$

Since the divergence of $\overrightarrow{F}$ is always negative (the parameters have to be always positive to make sense), the phase-space is not conserved and a 7-dimensional attractor exists. Further information on the stability of the system can be obtained if one fixes the values of the parameters in the equations. For example, with the set of parameters we used in our numerical experiments ($k_{m_1} = 23.5$, $k_{m_2} = 41$, $k_s = 17.5$, $k^{on}_{c_1} = k^{on}_{c_2} = 1.5$, $k_{p_1} = k_{p_2} = 117$, $\gamma_{m_1} = \gamma_{m_2} = 2$, $\gamma_s = \gamma_{p_1} = 1$, $\alpha\gamma_{c_1} = \alpha\gamma_{c_2} = 1.5$, $(1-\alpha)\gamma_{c_1} = (1-\alpha)\gamma_{c_2} = 1$, $h = 200$, $n = 1$), the system has a linearly stable fixed point.

7 Steady state analysis with the logic approximation

We discuss here the steady state analysis of the Eq.(1) of the main text in the framework of the logic approximation in which the Hill function is approximated with the Heaviside step function $f(p_1) = H(p_1 - h)$. Even if this approximation is very crude it may help to have an intuitive picture of the behaviour of the various players of the circuit as a function of the parameters of the circuit. The equations for p_1 and p_2 (see Eq.(1) of the main text) can be solved immediately leading to the steady state values: $p^0_1 = k_{p_1} m_1/\gamma_{p_1}$ and $p^0_2 = k_{p_2} m_2/\gamma_{p_2}$. We can rescale the activation coefficient $h_s \equiv h\gamma_{p_1}/k_{p_1}$ and then write the step function as a function of m_1 and eliminate p_1 from the equations. Following [16] we introduce the quantities ($i = 1, 2$)

$$\begin{aligned} \lambda_i &\equiv \frac{k^{off}_i + \gamma_{c_i}}{k^{on}_i}\,, \\ \theta_i &\equiv \frac{\gamma_{c_i}}{\gamma_{m_i}} M_{tot}\,, \end{aligned} \qquad (29)$$

which have an immediate physical interpretation: θ_i is the (suitably rescaled) amount of miRNA acting on m_i and $1/\lambda_i$ measures the strenght of this interaction, i.e. the lifetime of the complex c_i. These will be in the following the only external parameters of micFFL. Finally we assume for simplicity $\lambda_1 = \lambda_2 = \lambda$, $\theta_1 = \theta_2 = \theta$, and denote m^0_i as the steady state value m_i would reach if $M_{tot} = 0$ (i.e. $m^0_1 \equiv k_{m_1}/\gamma_{m_1}$ and $m^0_2 \equiv k_{m_2}/\gamma_{m_2}$ if $m^0_1 > h_s$ and $m^0_2 = 0$ otherwise). Then it is easy to obtain the steady state values of m_1 e m_2 as a function of θ and λ.

$$\begin{aligned} m_1 &= m^0_1 \frac{m^0_1 + m^0_2 - \theta - \lambda + \sqrt{\left((m^0_1 + m^0_2 - \theta - \lambda)^2 + 4(m^0_1 + m^0_2)\lambda\right)}}{2(m^0_1 + m^0_2)} \\ m_2 &= m^0_2 \frac{m^0_1 + m^0_2 - \theta - \lambda + \sqrt{\left((m^0_1 + m^0_2 - \theta - \lambda)^2 + 4(m^0_1 + m^0_2)\lambda\right)}}{2(m^0_1 + m^0_2)} \end{aligned} \qquad (30)$$

The implications of this result can be better appreciated if we take the $\lambda \to 0$ limit.

$$\begin{aligned} m_1 &= \begin{cases} 0 & m^0_1 + m^0_2 \leq \theta \\ m^0_1 \left(1 - \frac{\theta}{m^0_1 + m^0_2}\right) & m^0_1 + m^0_2 > \theta \end{cases} \\ m_2 &= \begin{cases} 0 & m^0_1 + m^0_2 \leq \theta \bigvee m_1 < h_s \\ m^0_2 \left(1 - \frac{\theta}{m^0_1 + m^0_2}\right) & m^0_1 + m^0_2 > \theta \bigwedge m_1 > h_s \end{cases} \\ &\text{with } m^0_2 = \begin{cases} 0 & m_1 < h_s \\ m^0_2 & m_1 > h_s \end{cases} \end{aligned} \qquad (31)$$

We plot the value of m_1 and m_2 as a function of θ (i.e. of the miRNA concentration) in fig.s 4a and 4b. Looking at these figures we see a few interesting and non trivial features:

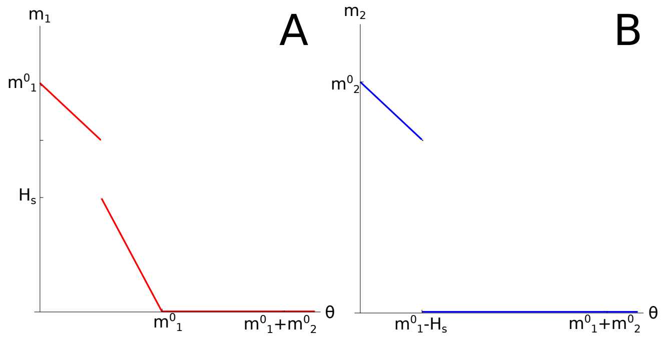

Figure 5. Steady state analysis with the logic approximation of the micFFL. Plots **A** and **B** show the mRNA concentrations, respectively, of transcription factor (m_1) and target (m_2) as a function of the microRNA concentration (θ) in the limit $\lambda \to 0$. H_s represents the activation threshold of the Heaviside function.

- In the $\lambda \to 0$ limit we find for the transcription factor m_1 the same threshold behaviour discussed in [16] as a function of the miRNA concentration. The same effect should be present also in the target concentration m_2, but is hidden by the fictious step behaviour due to the logic approximation. It is easy to understand the origin of this threshold behaviour: if the number of free miRNA molecules greatly exceeds the number of transcripts mTF and mT, then these will be almost all bound in complexes and the corresponding proteins will not be expressed. On the opposite side, if the number of mTF and mT molecules overcomes miRNA amount, then nearly all miRNAs will be bound in complexes but there will be a sufficient amount of free mTFs and mTs to be translated.

- As the total miRNA concentration decreases the TF concentration increases following the trajectory plotted in fig 5. When the TF concentration reaches the threshold h_s for the m_2 activation we observe a sudden enhancement in the TF concentration due to the sponge interaction between m_1 and m_2. When also m_2 is present then the two mRNAs start to compete for the same miRNAs and as a net effect there is a smaller amount of miRNA available to downregulate m_1. This non linear behavior of the TF concentration as the miRNA concentration increases is in our opinion one of the most effective ways to detect sponge-like interactions.

- The ratio m_2/m_1 (and thus p_2/p_1) can only take two possible values: $m_2/m_1 = m_2^0/m_1^0$ for $m_1 > h_s$ and $m_2/m_1 = 0$ for $m_1 < h_s$. However this is clearly an artifact of the logic approximation.
